# VANTED v2: a framework for systems biology applications

**DOI:** 10.1186/1752-0509-6-139

**Published:** 2012-11-10

**Authors:** Hendrik Rohn, Astrid Junker, Anja Hartmann, Eva Grafahrend-Belau, Hendrik Treutler, Matthias Klapperstück, Tobias Czauderna, Christian Klukas, Falk Schreiber

**Affiliations:** 1, Leibniz Institute of Plant Genetics and Crop Plant Research (IPK), Corrensstr. 3, 06466 Gatersleben, Germany; 2 Institute of Computer Science, Martin Luther University Halle-Wittenberg, Von-Seckendorff-Platz 1, 06120 Halle, Germany; 3 Clayton School of Information Technology, Monash University, Victoria 3800, Australia

**Keywords:** Biological networks, Data visualization, Data integration, Data analysis, -Omics, Model simulation

## Abstract

**Background:**

Experimental datasets are becoming larger and increasingly complex, spanning different data domains, thereby expanding the requirements for respective tool support for their analysis. Networks provide a basis for the integration, analysis and visualization of multi-omics experimental datasets.

**Results:**

Here we present Vanted (version 2), a framework for systems biology applications, which comprises a comprehensive set of seven main tasks. These range from network reconstruction, data visualization, integration of various data types, network simulation to data exploration combined with a manifold support of systems biology standards for visualization and data exchange. The offered set of functionalities is instantiated by combining several tasks in order to enable users to view and explore a comprehensive dataset from different perspectives. We describe the system as well as an exemplary workflow.

**Conclusions:**

Vanted is a stand-alone framework which supports scientists during the data analysis and interpretation phase. It is available as a Java open source tool from http://www.vanted.org.

## Background

Systems biology comprises the iterative cycling between experimental (wet-lab) and computational (dry-lab) approaches with the aim of generating a holistic understanding of biological systems. The complexity and comprehensiveness of experimental datasets is exponentially increasing thereby elevating the requirements for respective tool support. This motivates the development of adequate software solutions supporting the analysis, integration and visualization of multiple large-scale datasets.

The reconstruction of different kinds of networks (e. g., metabolic, signaling, protein interaction and gene regulatory networks [1]) based on experimental datasets allows for the representation of the diverse nature of biological systems on a global scale. Networks provide the basis for qualitative and quantitative network analysis, for example, for structural analysis and simulation. Networks can furthermore be used for the integrated visualization of multi-omics experimental datasets. In combination with exploration functionalities and further data analysis steps such as correlation and clustering this is crucial for the gain of knowledge from large-scale datasets. New insights lead to the generation of new hypotheses giving feedback to the wet-lab, thereby closing the knowledge generation cycle in systems biology.

To deal with technical advances and the consequent increase of genome-wide datasets, a number of very diverse tools has been developed for network-centered visualization and analysis of experimental data [2,3]. A tool supporting every step of the knowledge generation cycle has to provide the following functionalities: (1) import of data and networks as well as (2) the export of data analysis results and visualizations in different standardized file formats to utilize existing resources, communicate findings and distribute new knowledge among researchers, (3) a variety of analytical methods to extract novel biological findings from large-scale datasets thereby reducing the complexity of the dataset, (4) data integration to combine data from multiple data domains and support data analysis on a systems level and in the context of the ’global’ expertise, (5) model simulation to analyze the dynamic behavior and function of biological systems, thereby elucidating potential targets of biotechnological usage, (6) visualization to ease the understanding of complex datasets and help to elucidate previously unknown functional relations and (7) exploration and interaction functionalities to support visual analysis of large scale datasets and to adapt visualizations according to individual purposes.

Here we present Vanted (version 2) (hereafter named Vanted), a framework for systems biology applications, which emerged from the initial Vanted version [4]. Based on the previously described functionalities it comprises a comprehensive set of tasks ranging from network reconstruction, data visualization, integration of various data types, network simulation to data exploration combined with a manifold support of systems biology standards for visualization and data exchange.

**Figure 1:**
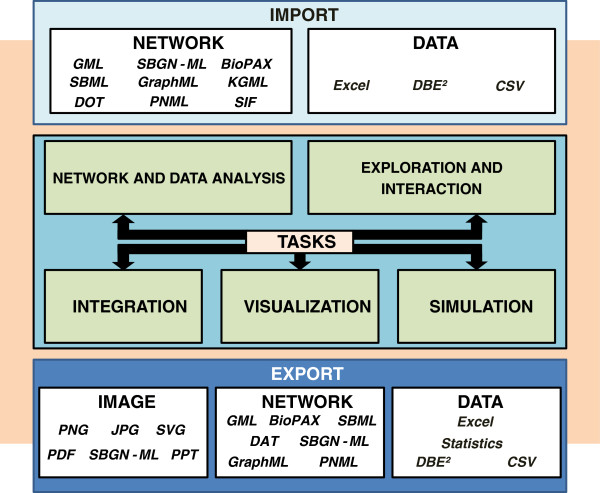
**Overview of tasks supported by VANTED.** After the initial import of network and experimental data, various tasks can be performed in a combinatorial fashion in order to instantiate a systems biology workflow. The export of results and visualizations is possible at each step of the workflow.

According to Figure 1 we will first introduce the seven main tasks of Vanted with a detailed explanation of various sub-tasks and indicate the possibilities for combining them in order to create systems biology workflows. In the second section an exemplary workflow is instantiated, demonstrating the combination of sub-tasks in order to explore a complex metabolite dataset. Finally, we discuss the benefits of the Vanted framework and describe potential future use cases and corresponding developments of the system.

## Implementation

The initial Vanted framework was published in 2006 [4] and is widely used throughout the biologists community (see, for example, [5-11]). In the last years, the framework has been substantially extended and the structure has been changed by out-sourcing of sub-tasks from the Vanted core into add-ons, which are functional modules that can be added during run-time (see Table 1). Such modular approaches allow for a stable and easily maintainable framework core while enabling users to compose a set of functionalities according to individual purposes (see [12,13] for other examples). Vanted has been extended by several important technical improvements such as identifier enrichment for network elements, new input and output interfaces, self-organizing map clustering (SOM)[14], KEGG editor functionality [15] and many more. The new Vanted framework provides a diverse set of functionalities which support system biologists in visualizing and analyzing large-scale datasets (see Figure 1). These can be roughly categorized into seven main tasks, explained in the following sections and Table 1. 

**Table 1:** Summary of tasks supported by VANTED

**Task**	**Sub-Tasks**	**Implemented in**
Import	∙ networks (GML, GraphML, SBML, KGML, SIF,	
	DOT, BioPAX, SBGN-ML, PNML)	core, MetaCrop add-on, DBE^2^ add-on
	∙ experimental data (XLS, XLSX^∗^, CSV)	
	∙ connection to experiment database DBE^2^	
	∙ connection to network databases	
	(MetaCrop, KEGG, RIMAS)	
Visualization	∙ charts (line, bar, pie, heat maps) on nodes and edges	core, SBGN-ED add-on,
		HIVE add-on, FluxMap
		add-on
	∙ automatic network layouts (e. g., Graphviz,	
	force-directed, tree layout)	
	∙SBGN support	
	∙flux data support	
	∙3D visualization of networks and multimodal data	
Integration	∙mapping of numerical or multimodal data	
	∙mapping tables, identifier mapping	
	∙ linking other resources	core, HIVE add-on
Simulation	∙constraint-based analysis	FBA-SimVis add-on,
	∙Petri net analysis	Petrinet add-on
Exploration and interaction	∙ panning, zooming, collapsing, search, selection	
	∙network exploration	
	∙brushing, image exploration	core, GLIEP add-on, HIVE add-on
Analysis	∙ networks (centralities, shortest path, cycle detection, motifs)	core, Centilib add-on
	∙ statistics (correlation, clustering, t-test)	
	∙enrichment analysis	
Export	∙ raster graphics (PNG, JPG), vector graphics	
	(SVG, PDF, PPT, SBGN-ML)	core, DBE^2^ add-on
	∙interactive websites	
	∙ experimental data (XLS, XML, DBE^2^)	
	∙ networks (GML, GraphML, DAT (Metatool),	
	SBML, SBGN-ML, PNML)	

The first column comprises the task covered by the Vanted framework. The second column shortly summarizes sub-tasks. Underlined sub-tasks indicate new functionalities developed since the initial Vanted publication in 2006 [4]. The third column lists the modules of the Vanted framework (Vanted core, add-ons) that implement the described tasks.

### Import

Common network exchange formats are supported such as SBML [16], BioPAX [17], KGML [18], GML [19], DOT [20], SBGN-ML [21] and SIF [12] thereby enabling the exchange of data throughout the community. Various databases (e.g., KEGG [22]) provide network files which can be imported into Vanted via drag-and-drop. Vanted is directly connected to the MetaCrop and the RIMAS databases. The MetaCrop database [23] contains manually curated information about metabolic pathways of major crop plants and corresponding networks in SBGN [24]. In addition to metabolic pathways the database comprises information about reaction kinetics and gene identifiers as well as related literature references. In order to filter, explore and import this information, the MetaCrop add-on provides seamless access [25]. Besides metabolic networks, gene regulatory networks of the RIMAS web portal [26] can be directly accessed. This information resource comprises SBGN-style networks about regulatory interactions during seed development of *Arabidopsis thaliana*.

The import of experimental data is preferably done by using XLS templates, which enable a structured import together with meta-data. Alternatively, plain text or CSV files may be used to import large datasets such as gene expression data, but require manual enrichment with meta-data. For unlimited accessibility, persistent storage and exchange of experimental data, the DBE^2^ information system [27] is accessible via the DBE^2^ add-on. The add-on utilizes ontologies from the Ontology Lookup Service [28] to unify terms such as compound names, species names and measurement units aiming at a facilitated data integration. As Vanted, DBE^2^ supports different data types from numerical data to images, three-dimensional volumes and networks.

### Visualization

Networks are represented as graphs composed of nodes and edges with fully customizable visual appearance. Numerous visual attributes such as the position, size, color and frame thickness of nodes as well as the color and thickness of edges and other visual attributes such as labels can be adapted according to individual purposes. In addition, a specialized set of node and edge shapes is provided, which build the basis for an SBGN compliant network visualization. SBGN-ED [29] enables Vanted to adapt networks for all SBGN languages in order to facilitate a standardized visual representation of biological entities. The visualization of such maps can be validated for syntactic and semantic correctness according to the SBGN specification.

Readable network layouts are important to improve the visual representation of networks. Besides the manual layout of network elements, automated graph layout algorithms are provided by calling the external Graphviz layouter API [30] or executing self-implemented layouters based on Tollis *et al*. [31] such as the force-directed layout, tree layout, circle layout, expression matrix layout, grid layout, subgraph layout and edge-routing algorithms. Further editing or improvement of automatic layouts can be done by manual curation using node merging and splitting algorithms. The latter is important for splitting frequently occurring nodes such as ATP or CO_2_ in metabolic networks, thereby preventing edge-crossings throughout the network.

Vanted offers the integration of various datasets into network nodes and edges (data mapping) thereby enabling a network-based view on large-scale datasets. Options for visual representation of experimental data include shape and color coding of nodes and edges as well as more complex visualizations such as bar charts, pie charts, line charts and heat maps. Experimental factors of complex datasets such as time-resolution, varying genotypes and environmental conditions can be represented within one chart. Visualization of charts is performed by calling the JFreeChart library [32]. The FluxMap add-on [33] enables the visual representation of flux data by edge thickness adaptation. This supports the comparative visual analysis of complex flux distributions in an interactive way. Using the HIVE add-on [34] image-based data such as histological cross-sections, microscopy images, photographs and three-dimensional volume data such as NMR and CT data can be displayed in the network context based on a workspace approach and rendered using various 2D-, 3D- and network visualization functions.

Every shape, label, chart and even the selection are realized in Vanted as single Java Swing components placed in the graph window (for further technical details see [35]). Other commonly used libraries such as JUNG[36] render all graphics in a single component. Vanteds approach is harder to implement, but scales better in terms of rendering speed and enables high flexibility in adapting and fine-tuning each component. The highly optimized Cytoscape framework on the other hand scales very good, but does not enable comparable flexibility in terms of visualization of charts, shapes and other graphics.

In general, visualization is the most advanced feature of Vanted. Multiple options and functionalities enable users to generate appropriate visual representations thereby substantially facilitating the gain of knowledge compared to working with data tables. Vanted enables users to interact with up to 10k network elements, but the responsiveness depends on the visual complexity as complex charts, labels and other visualizations as well as high numbers of edge crossings may reduce this numbers considerably down to some thousand elements. For larger graphs, interaction may become unfeasible and algorithms such as automatic layouters consume a considerable amount of time.

### Integration

Biological entities such as proteins, genes or metabolites are represented as nodes and any relation between such entities as node-connecting edges (e.g., regulation, interaction or conversion). Both network elements are attributed by technical properties such as visualization parameters (size, position, etc.) and properties related to their biological role. Each network element may contain links to other resources, usually represented as a hyperlink to any web-content such as a database entry. Nodes may link to other networks, enabling navigation and exploration of connected pathways (see also Section Exploration and interaction). Based on the present numerical attributes, for example, size, position and node degree, the user is able to compute new properties such as additional median values, which are stored as new element attributes and may be visualized or exported.

In Vanted, network elements are allowed to have several (alternative) identifiers. These identifiers provide the basis for data mapping which depends on common identifiers in network and experimental data. In case of different identifiers, synonyms have to be defined. For this mapping tables may be used to provide either additional labels for network elements or for biological entities in the experiment data. Mapping tables are simple XLS files, which list the existing names in the first column and additional names in the subsequent columns.

### Simulation

Basis of the simulation task is the modeling capability of Vanted. Model reconstruction is based on a given network topology, which is manually created or imported from network files. Subsequently, model attributes such as stoichiometric coefficients, kinetic constants, firing rules and initial markings are added to the network or are already part of the import process (SBML files for example provide most attributes). So far, Vanted does not support the automated reconstruction of networks from external sources as described in [37].

These biological networks are finally transformed into mathematical models in order to analyze dynamic properties and behavioral attributes. The enrichment of metabolic networks with stoichiometric coefficients (represented by edge weights) and the definition of an optimization function is a prerequisite for the constraint-based network analysis. The FBA-SimVis[38] add-on enables Vanted to perform different techniques such as Flux Balance Analysis [39], Flux Variability Analysis [40], Robustness Analysis [41] and Knock-out Analysis. In combination with a dynamic and visual exploration of simulation results, this allows for the comprehensive analysis of metabolism in response to genetic or environmental perturbations. Metabolic networks can also be transformed into Petri nets [42], a second mathematical model, which is used for formal analysis and simulation of biological systems. The Petrinet[43] add-on enables Vanted to semi-automatically transform networks into valid Petri nets, simulate discrete and continuous Petri nets of varying complexity and analyze structural properties. Different visualization and interaction techniques such as brushing can be utilized in order to visually analyze P- and T-invariants, the reachability graph and varying markings of simulation steps.

### Exploration and interaction

In terms of exploration of networks and data visualizations, Vanted supports standard interaction methods such as panning, zooming and overview+detail for selected network elements. The editing and rearrangement of network elements as well as the modification of attribute values and calculation of new attributes is possible in an interactive manner. Sophisticated selection and search functionalities provide the ability to find and explore network elements based on attribute values.

Furthermore, recurring entities in large networks or several networks may be linked in order to easily track interconnections between pathways. The GLIEP[44] add-on provides an interactive view for the exploration of interconnected networks by implementing a glyph visualization. Based on these glyphs the user is able to quickly switch between connected networks or to explore the overall interconnectivity using a focus+context technique. Furthermore, the HIVE add-on enables users to collapse networks into single nodes, thereby providing a clear representation of multiple (interconnected) networks. Connections between different networks are retained and link the network-overview nodes, which can be re-arranged or expanded according to user requirements.

On the basis of interaction events such as selection, brushing techniques [45] provide different views on visualized experimental data. The HIVE add-on enables users to explore and compare spatial distributions within a biological system by parallel visualization of segmented images and experimental values in the network view. Hovering over a segment in the image (e.g., corresponding to an organ) results in highlighting the respective measurement values in the network view. Furthermore it is possible to explore large numbers of images in the context of a network. If these images are related to a substance (e.g., GFP reporter expression for genes in a gene regulatory network), the user can integrate the respective images into the network nodes. If a number of nodes is selected, an image matrix is built up, spanning conditions, time points and replicate information. This matrix enables users to compare all images related to the selected nodes and to explore spatial patterns of different substances in the context of a biological network.

Further brushing techniques are provided by the Petrinet add-on for the analysis of Petri net properties such as invariants and the reachability graph. The user can move the mouse over nodes of the reachability graph, triggering the visualization of the respective state in the network visualization view.

### Analysis

The analysis of network topology plays an important role for the understanding of interactions between biological entities. Vanted offers to compute several topological properties such as shortest paths between node pairs, network cycles and motifs. The detection of network motifs (such as feed-forward loops) is supported by the possibility to search for user-defined motifs which might be meaningful in the context of certain biological questions. The Vanted add-on Centilib[46] provides algorithms and methods for the computation and investigation of 17 different centralities in biological networks. Such centralities can be used for ranking of network nodes according to given criteria and for the detection of network hubs. Results of the centrality analysis can be explored and analyzed using a brushing-based approach.

The statistical evaluation of experimental datasets is a central part of data analysis. Vanted offers a series of tests for calculation of statistical parameters, for testing the normal distribution of datasets (David Quicktest [47]) and for outlier detection (Grubbs test). For the comparison of measurements with multiple conditions, several t-tests are available such as the unpaired t-test, the Welch-Satterthwaite t-test and the Mann-Whitney U-test with user-defined threshold settings for the calculated p-values. Vanted enables users to perform Pearson’s and Spearman correlation analysis based on the mapped experimental data. Optional settings include a p-value threshold and the number of experiment conditions included in the analysis (see [4] for implementations details).

The calculation of clusters is a frequently used approach to categorize experimental data into functional or behavioral groups. For this task, Vanted supports self-organizing maps (SOM) [14]. A SOM is an artificial neural network, which is capable for the automated recognition of patterns within measurements and is well-suited for the categorization of time series data of biological entities. According to a user-defined number of target clusters, the SOM is trained and cluster attributes are automatically assigned to the network nodes. In addition such assignments can be done manually. The cluster sub-networks may then be independently laid out or colorized in order to visually catch clustered elements at a glance.

For gene expression data Vanted supports the computation and visualization of enrichments in the context of the GO [48] and the KEGG pathway [22] hierarchies. For example, for KEGG the procedure highlights classes of KEGG pathways in which the experimental data enriches significantly by assigning pie charts [49,50].

### Export

Vanted provides a variety of file formats for data storage, publication and exchange. The GML and GraphML file formats are Vanted s native formats and accordingly support the storage of networks together with all related attributes such as layout information and the full set of mapped and integrated experimental data including the visualization options for mapped data. Additional information can be stored and exchanged as new attributes, e. g. a new custom attribute “myAttribute” enables to colorize all nodes with this attribute based on the respective attribute value. Such attributes can be created manually (e. g. cluster information and biological tags) or be the result of a computation (see [35] for further details).

For the exchange of data within the systems biology community, support for file formats such as DAT [51], SBGN-ML (provided by the SBGN-ED add-on) and BioPAX is implemented. Vanted additionally supports the SBML file format which allows for the storage and exchange of stoichiometric and kinetic models. When working with the Petrinet add-on, the Petri net and its configuration can be exchanged using the PNML file format. Experimental data which has been mapped onto a network can be extracted and exported using XLS sheets. The CSV format is supported for different kinds of node attributes as well as the export of analysis results such as correlation coefficients. All data types which are supported by Vanted (numerical data, images, three-dimensional volumes, networks) can be uploaded to the DBE^2^ system for persistent data storage and exchange. Please note that Vanted usually serves as a data sink and the conversion between different file formats is not in the focus of the tool. Network topology (including labels) on the other hand is preserved in most cases.

Laid out networks can be exported to several graphic file formats, including raster images (PNG, JPG), as well as vector images (SVG, PDF, PPT). These file formats are well suited to be used as images in publications, presentations or as a basis for further graphical editing. Furthermore it is possible to export integrated networks as browseable and clickable images, embedded in HTML web sites. Those images can contain web-links to web resources or public databases. The publishing process of these web sites can be done in a semi-automatic fashion [52].

## Results

**Figure 2:**
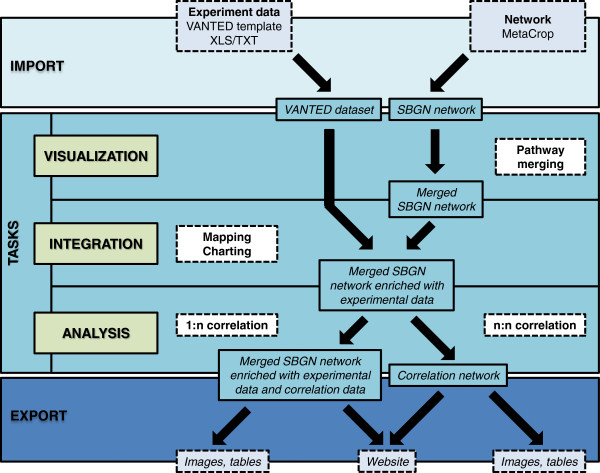
**VANTED workflow for the exemplary use case.** A complex metabolite dataset is imported into Vanted, integrated and visualized in the context of a large SBGN-style metabolic network. Based on data mapping, different kinds of correlation analyses are performed. The results of the workflow can be exported in various formats.

The previously described tasks can be instantiated and combined in order to create manifold workflows supporting the interpretation of systems biology data. For demonstration purposes an exemplary workflow is executed with the Vanted framework, implementing the analysis of a comprehensive metabolic dataset taken from Sulpice *et al.*[53]. This dataset consists of measurements of enzyme activity data, metabolite data and different morphological parameters for a wide range of *Arabidopsis thaliana* ecotypes. In the following we focus on the first ecotype class A, which includes the most diverse ecotypes. The steps of the workflow are depicted in Figure 2 and the tutorial (Additional file 1). 

### Import

The import of enzyme activity data, metabolite data and morphological parameters of different *Arabidopsis thaliana* accessions from climate class A is realized using the Vanted XLS template (see Additional file 2). Experimental data may also be persistently stored in the DBE^2^ database, enabling file sharing and on-click import of such experimental data into Vanted. In parallel to the import of the experimental data, 38 metabolic reference pathways are loaded from the MetaCrop database and merged into one SBGN network. Subsequently all reference pathways are assigned to their respective cellular location and the pathways in each subcellular compartment are connected to each other by merging identical metabolite nodes. Finally a network layout is performed in order to optimize the edge routing and distance between nodes, resulting in the network which can be found in Additional file 3.

### Visualization and integration

**Figure 3:**
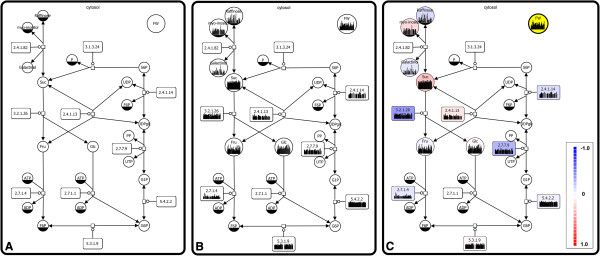
**Visualization, integration and analysis of plant metabolic networks. (A)** Metabolic network representing sugar metabolism in SBGN. A new node for the morphological parameter fresh weight (FW) was added to the network. **(B)** Integration of metabolic data into the network by visualization of corresponding charts inside the nodes. Metabolite concentrations are mapped to simple chemical glyphs whereas enzyme activity data is mapped to macromolecule glyphs. Bar charts display respective values for all *Arabidopsis thaliana* accessions of climate class A. **(C)** 1:n correlation analysis on mapped data for the detection of correlations between the morphological parameter FW and all other metabolic parameters. Correlation coefficients are visualized by color-coded nodes.

During data mapping, experimental data is integrated into the network by the visualization of corresponding charts inside the network nodes. To unify the identifiers in the network and the experimental dataset, a mapping table is used for the enrichment of network nodes with alternative identifiers (Figure 3a and Additional file 3). Subsequently, metabolite data is mapped to the nodes representing metabolites (simple chemical glyph) and enzyme activity data is mapped to nodes representing enzyme nodes (macromolecule glyph). New nodes for morphological parameters are added during the mapping process, as they are part of the experimental data, but do not occur in the network. The mapped experimental data is visually represented by bar charts inside the glyphs resulting in a data-enriched SBGN network (Figure 3b and Additional file 4).

### Analysis

In order to identify similarities in the profiles of all accessions of climate class A, 1:n and n:n correlation analyses are performed. In case of the 1:n correlation analysis, the morphological parameter fresh weight (FW) is chosen as the target parameter and correlations were calculated to all other metabolic parameters in the network. Based on the resulting correlation coefficients network nodes are color-coded according to the correlation coefficient *r* (Figure 3c and Additional file 5). This visual representation of correlation results enables biologists to easily identify metabolic parameters with important influence on plant morphology at a global scale.

**Figure 4:**
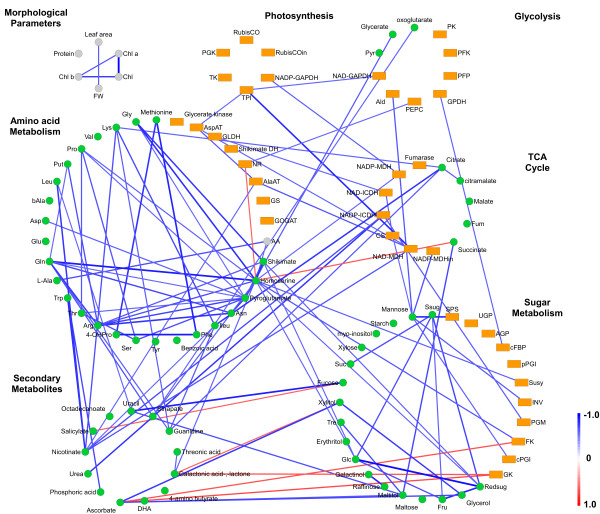
**Correlation network for different pathways.** Nodes representing metabolites (green), enzymes (orange) and morphological or other parameters (gray) are laid out as circles for each pathway. A n:n correlation was calculated, resulting in edges indicating a strong (*p*≥0.95) correlation, color-coded by the *r*-value. This visualization enables an overview about intra- and inter-pathway dependencies.

For the n:n correlation analysis, all metabolic parameters in the network are correlated with each other, including all metabolite and enzyme activity data as well as the data of morphological parameters. The resulting correlation values are visualized by generating new edges between correlating nodes. These edges are color-coded according to the negative (red) or positive (blue) correlations calculated with *p*≥0.95 and |*r*|≥0.6 Pearson’s product-moment correlation. The resulting network is used to generate a correlation network at a pathway level, independent of the order of metabolic reactions within a pathway. Consequently, the metabolic dataset is used to generate new nodes in a network-independent manner which are then categorized according to the metabolic pathway (e.g., Glycolysis, TCA cycle) and laid out as pathway-specific circles (see Figure 4). During the n:n correlation analysis Vanted generates edges between nodes with data profiles of significant similarity thereby giving an overview about intra- and inter-pathway dependencies and allows for drawing conclusions about the interaction between single parameters. For example, the levels of amino acids show strong positive correlations among each other and with levels of TCA cycle intermediates, as these substances are precursors of the amino acids. This leads to the assumption that these mentioned parts of primary metabolism are stable throughout the different ecotypes. Secondary metabolites show strong negative correlations with enzymes of sugar metabolism among the considered *Arabidopsis thaliana* accessions. Variations of the levels of plant secondary metabolites are conceivable for accessions with different origin.

## Discussion

The Vanted framework provides a rich variety of functionalities at the interface between data analysis, gain of knowledge out of large-scale datasets and the generation of feedback to the wet-lab part of the systems biology cycle. It supports both the fast and customizable visualization of networks and experimental data as well as the exploration, simulation and different kinds of data analysis. In contrast, most network-centered tools focus on a small subset of tasks (compare Table 2). For instance, Omix provides high-quality and customizable network visualization but lacks analysis algorithms and direct connection to important databases. Ondex focuses on the generation of large-scale biological networks from heterogeneous sources, but does not support charts and simulations. CellDesigner is designed for the analysis of the dynamics of metabolic models, but does neither provide statistical analysis nor advanced interaction techniques. Vanted combines these features in one framework thereby reducing the use of several tools and tedious file exchanging procedures.

**Table 2:** Comparison of non-commercial tools for the network-centered visualization and analysis of biological data

**Tasks**	**Vanted**	**Cytoscape**	**Ondex**	**Omix**	**Cell**	**PathVisio**	**BioUML**	**VisAnt**	**Pathway**	**BiNA**	**MapMan**
		**[**[12]**]**	**[**[54]**]**	**[**[55]**]**	**Designer**	**[**[57]**]**	**[**[58]**]**	**[**[59]**]**	**Projector**	**[**[61]**]**	**[**[62]**]**
					**[**[56]**]**				**[**[60]**]**		
Import											
networks	+	+	+	+	+	(+)	+	(+)	+	+	-
experimental data	+	+	+	+	(+)	+	+	-	+	(+)	+
connection to experiment database	+	+	+	-	(+)	-	-	-	-	+	-
connection to network databases	+	+	+	-	(+)	-	+	+	(+)	+	+
Visualization											
charts on nodes and edges	+	-	-	+	-	-	-	-	+	-	+
automatic network layouts	+	+	+	+	+	-	+	+	-	+	-
SBGN support	+	+	-	-	(+)	+	+	-	-	-	-
flux data support	+	+	-	+	+	-	-	-	(+)	+	-
3D visualization	+	(+)	-	+	-	-	-	-	-	-	-
Integration											
mapping of numerical or	+	+	+	+	+	+	-	-	+	+	+
multimodal data											
mapping tables, identifier mapping	+	+	+	-	-	(+)	+	-	+	(+)	-
linking other resources	+	+	+	+	+	+	+	+	+	-	+
Simulation											
constraint-based analysis	+	(+)	-	+	(+)	-	-	-	-	-	-
Petri net analysis	+	-	-	-	(+)	-	-	-	-	-	-
Exploration and interaction											
panning, zooming, collapsing,	+	+	+	+	(+)	(+)	+	+	+	+	(+)
search and selection											
network exploration	+	+	+	-	-	(+)	+	+	+	+	+
brushing, image exploration	+	-	-	-	-	-	-	-	-	-	-
Analysis											
networks	+	+	+	-	(+)	-	+	+	-	(+)	-
statistics	+	+	+	-	-	+	-	-	-	(+)	+
enrichment analysis	+	+	-	-	-	+	+	+	-	+	+
Export											
raster graphics, vector graphics	+	+	+	+	+	+	+	+	-	+	(+)
interactive websites	+	-	-	-	-	(+)	+	+	+	-	-
experimental data	+	+	-	-	+	-	+	-	+	+	-
networks	+	+	+	+	+	+	+	(+)	-	+	-

The first column comprises the sub-tasks of Table 1, which are covered by the respective tool. Please note that also add-ons and plugins of the respective system were evaluated. “-” no or inadequate support, “(+)” = partial support, “+” good support of the sub-task.

Cytoscape is a widely used biological network analysis tool, which is the only competing tool providing all tasks in one system. Both tools cover a large portion of important systems biology tasks. Cytoscape lacks some functions such as sophisticated charts and website export, but compared to Vanted provides additional functionality which is usually not in the focus of systems biology researchers, such as social graph topics. It has a big developer community which implemented a large number of plugins (over 150). Although the sheer number of extensions is quite impressive, the quality and complexity varies significantly. Many Cytoscape plugins only provide simple functionalities such as the import of a certain file format, whereas others focus on very special applications which are not in the scope of the majority of potential users. In comparison to Cytoscape, the Vanted add-on concept relies on a smaller set of add-ons each comprising a large set of functionalities which are necessary in order to perform a whole workflow. Many Vanted add-ons are able to interact with each other, thereby increasing the capabilities of the core tool. Examples for such combinations are the HIVE and the DBE^2^ add-on, which together enable the persistent storage of volumetric and image data in the exchange database. Also the combination of FluxMap and SBGN-ED enables the visualization of flux data in SBGN networks. In summary, Vanted and Cytoscape both enable the execution of various systems biology tasks within one tool. Cytoscape provides a larger set of special sub-tasks with varying quality, whereas Vanted provides a small set of sub-tasks, which are optimized with regard to solving specific biological questions.

## Conclusions

Vanted is a stand-alone framework which supports scientists during the data analysis and interpretation phase. This is achieved by integrating experimental data into biological networks and providing a rich variety of simulation, analysis and visualization functionalities. Manifold file exchange formats as well as connections to databases enable the examination of user data in the context of public resources. In comparison to other tools Vanted provides a large variety of functionalities, spanning most of the tasks during the analysis and visualization of large-scale datasets. The offered set of functionalities enables users to view and explore data from different perspectives, thereby facilitating the systemic analysis of a biological object. The support of various standards enables users to easily exchange files using well-established standard file formats and allow for an accurate exchange of biological information using an unambiguous graphical representation (SBGN). To deal with future user requirements the Vanted system can be extended in a flexible way by using BeanShell and JRuby scripts or by writing new add-ons.

In the future we expect novel use cases to emerge for the Vanted framework, especially large datasets spanning multiple biological levels such as gene expression, protein activity, metabolite, flux and phenotypic data from one biological system [63]. Furthermore, the spatial resolution of the analyzed systems (e.g., compartmentation, tissues and organs) increases based on technological advances and enhanced quantity and quality of imaging techniques. Finally, mathematical models become more important for the understanding and prediction of complex behavior of biological systems.

## Availability and requirements

**Project Name:**Vanted

**Project home page:**http://www.vanted.org

**Operating system(s):** Platform independent (Java), the add-on FBASimVis will work on Windows computers only

**Programming language:** Java 6/7

**License:** GPL 2.0

## Competing interests

The authors declare that they have no competing interests.

## Authors’ contributions

CK, HR and TC implemented the core. HR, HT, EGB, TC and MK implemented the add-ons. AJ, AH, EGB and HR developed the use case. FS supervised the project and gave conceptual advice. HR wrote the manuscript; all authors contributed to, read and approved the manuscript.

## Supplementary Material

Additional file 1 **Supplementary tutorial.** ZIP file containing the data for recreating Figures 3 and 4. To guide the user, a PPT file is provided, which lists and describes all necessary steps to be performed in Vanted. Click here for file

Additional file 2 **Filled experiment data template.**Vanted template filled with metabolite data from Sulpice *et al.*[53], consisting of 64 metabolites, 37 enzymes and morphological parameters for 50 *Arabidopsis thaliana* ecotypes of climate class A. The file can be opened using MS Excel and imported into Vanted as an experiment dataset. Click here for file

Additional file 3 **Merged SBGN network.** Large-scale metabolic network of plant primary metabolism in SBGN. The network has been created with Vanted based on merging different pathways downloaded from MetaCrop. This file serves as the basis for mapping experiment datasets and can be imported into Vanted as a network. Click here for file

Additional file 4 **Merged SBGN network enriched with experimental data.** Enriched metabolic SBGN network after mapping additional file 2 onto additional file 3. Metabolite data of 50 *Arabidopsis thaliana* ecotypes is mapped to the network and visualized as bar charts inside the nodes. This file can be imported into Vanted as a network. Click here for file

Additional file 5 **Merged SBGN network enriched with experimental data and correlation data.** Analysis of enriched metabolic SBGN network by performing a 1:n correlation between the morphological parameter fresh weight (FW) and all enriched network nodes. The correlation coefficient is visualized using a global color-code. This file can be imported into Vanted as a network. Click here for file
