# Supplementary material for: VANTED v2: a framework for systems biology applications
Source: BMC Syst Biol. 2012 Nov 10;6:139. doi: 10.1186/1752-0509-6-139 (PMC3610154; doi:10.1186/1752-0509-6-139)
Supplement: Additional file 1 — Supplementary tutorial. ZIP file containing the data for recreating Figures 3 and 4. To guide the user, a PPT file is provided, which lists and describes all necessary steps to be performed in Vanted. [file 1752-0509-6-139-S1.zip › Tutorial.pptx]

## Slide 1
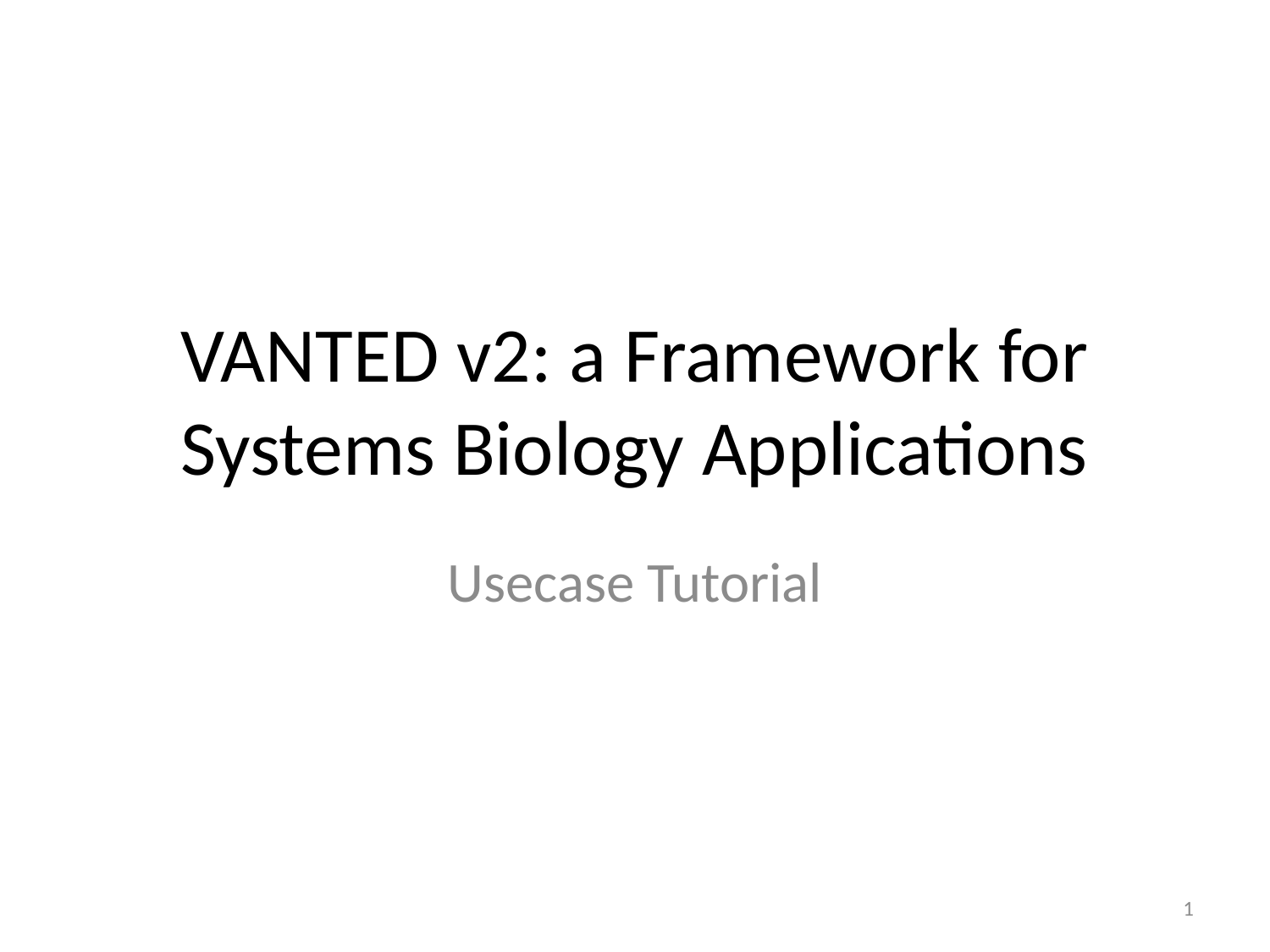

# VANTED v2: a Framework for Systems Biology Applications
Usecase Tutorial
1

## Slide 2
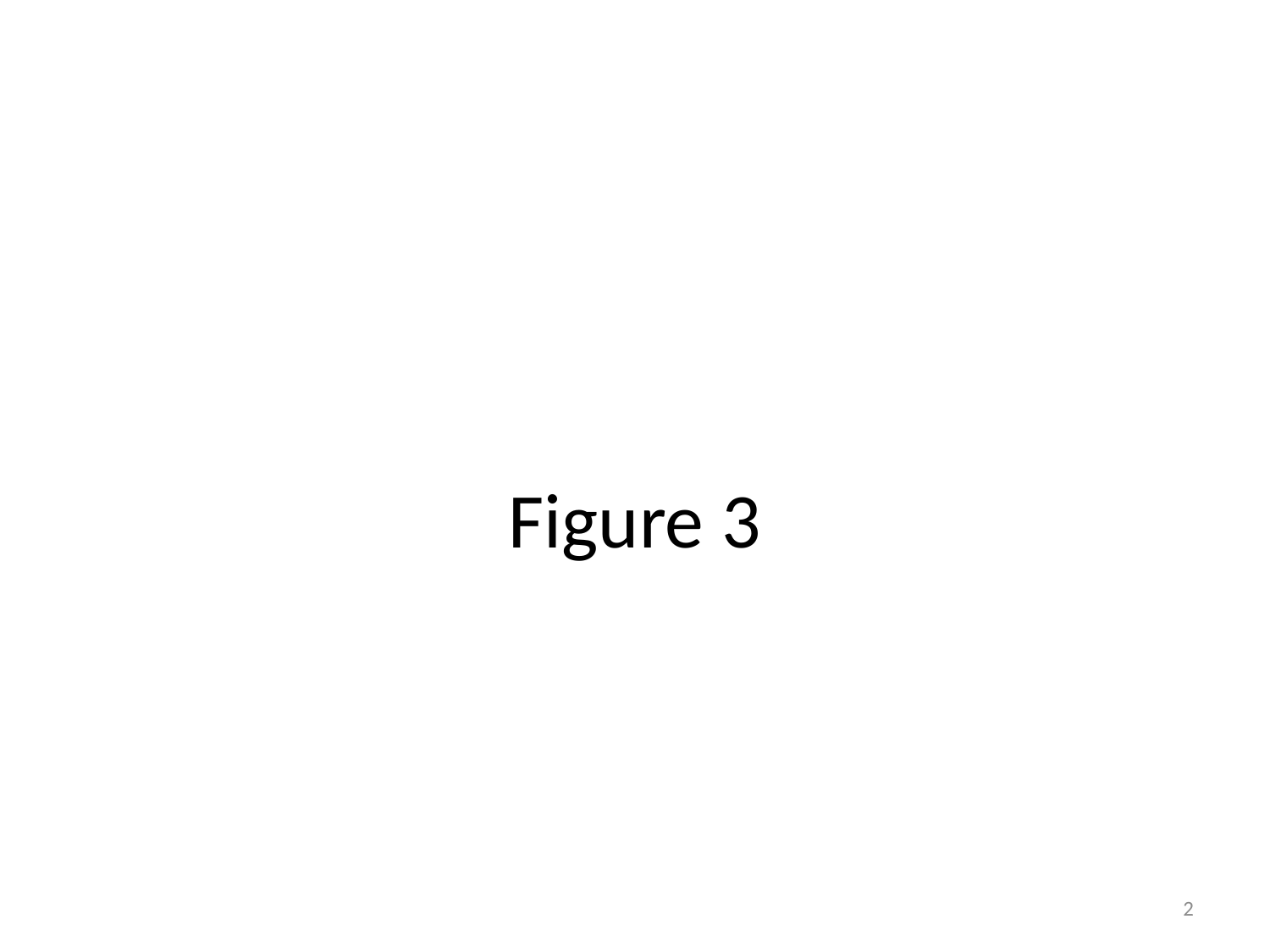

# Figure 3
2

## Slide 3
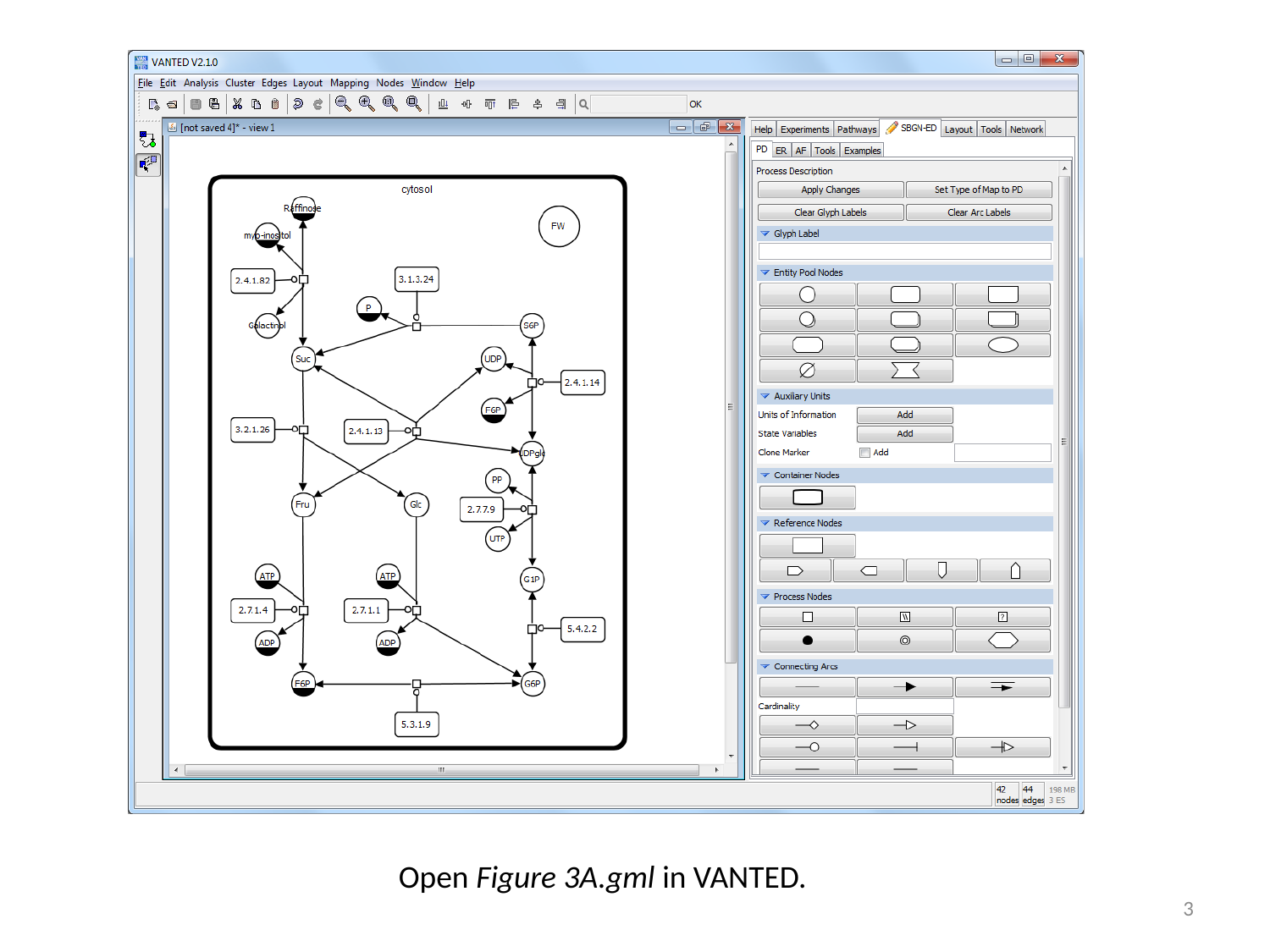

Open Figure 3A.gml in VANTED.
3

## Slide 4
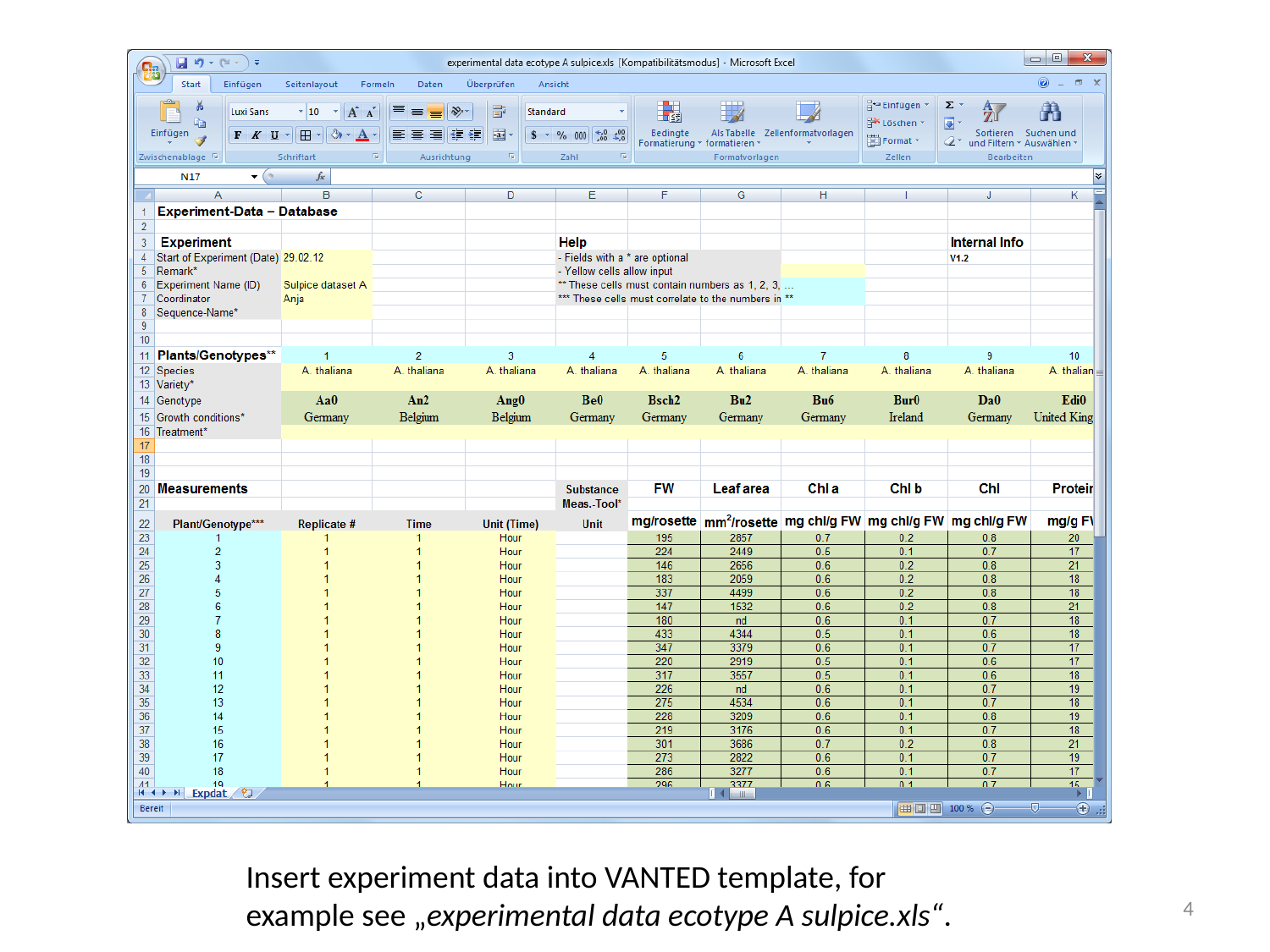

Insert experiment data into VANTED template, for example see „experimental data ecotype A sulpice.xls“.
4

## Slide 5
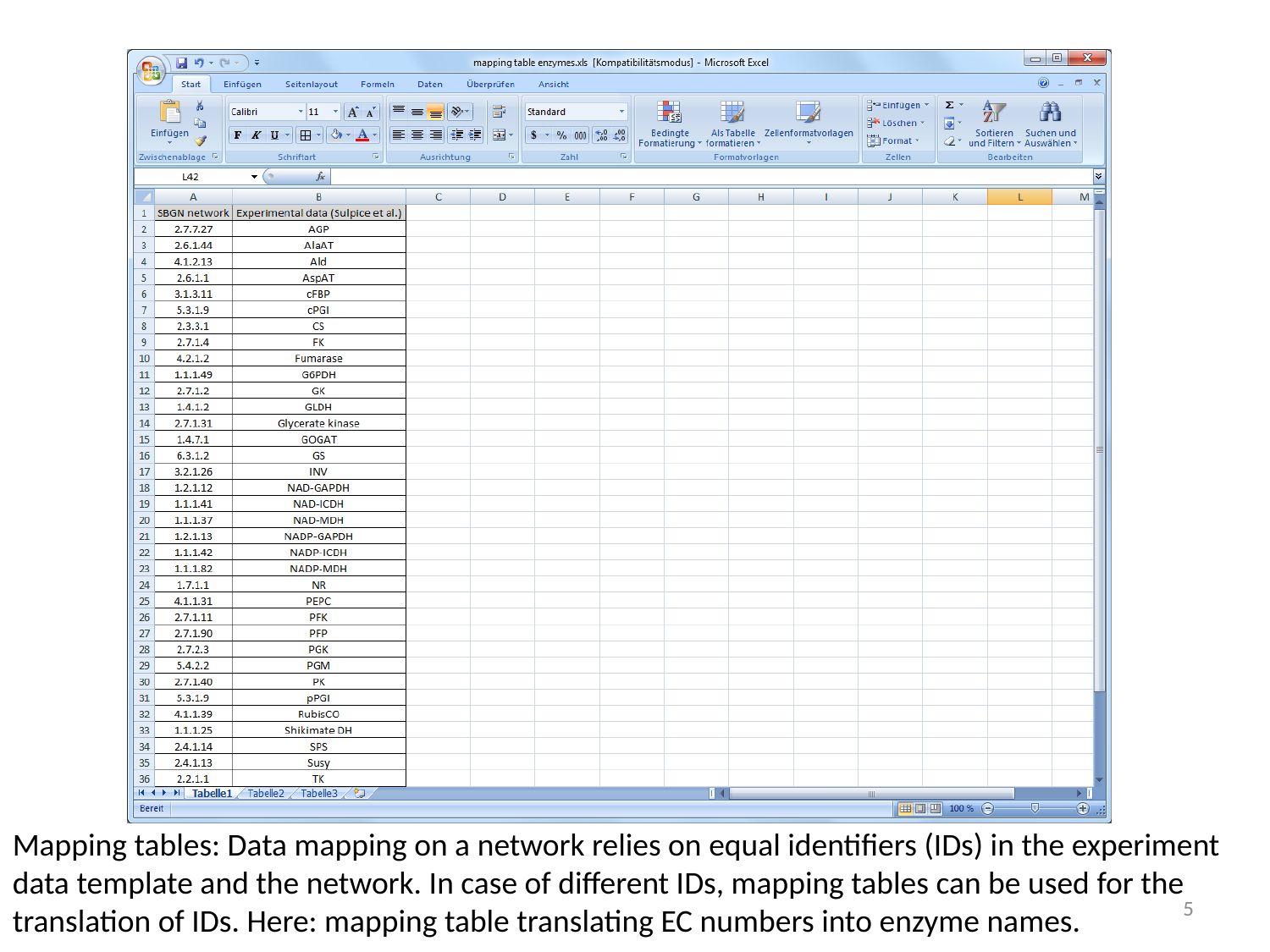

Mapping tables: Data mapping on a network relies on equal identifiers (IDs) in the experiment data template and the network. In case of different IDs, mapping tables can be used for the translation of IDs. Here: mapping table translating EC numbers into enzyme names.
5

## Slide 6
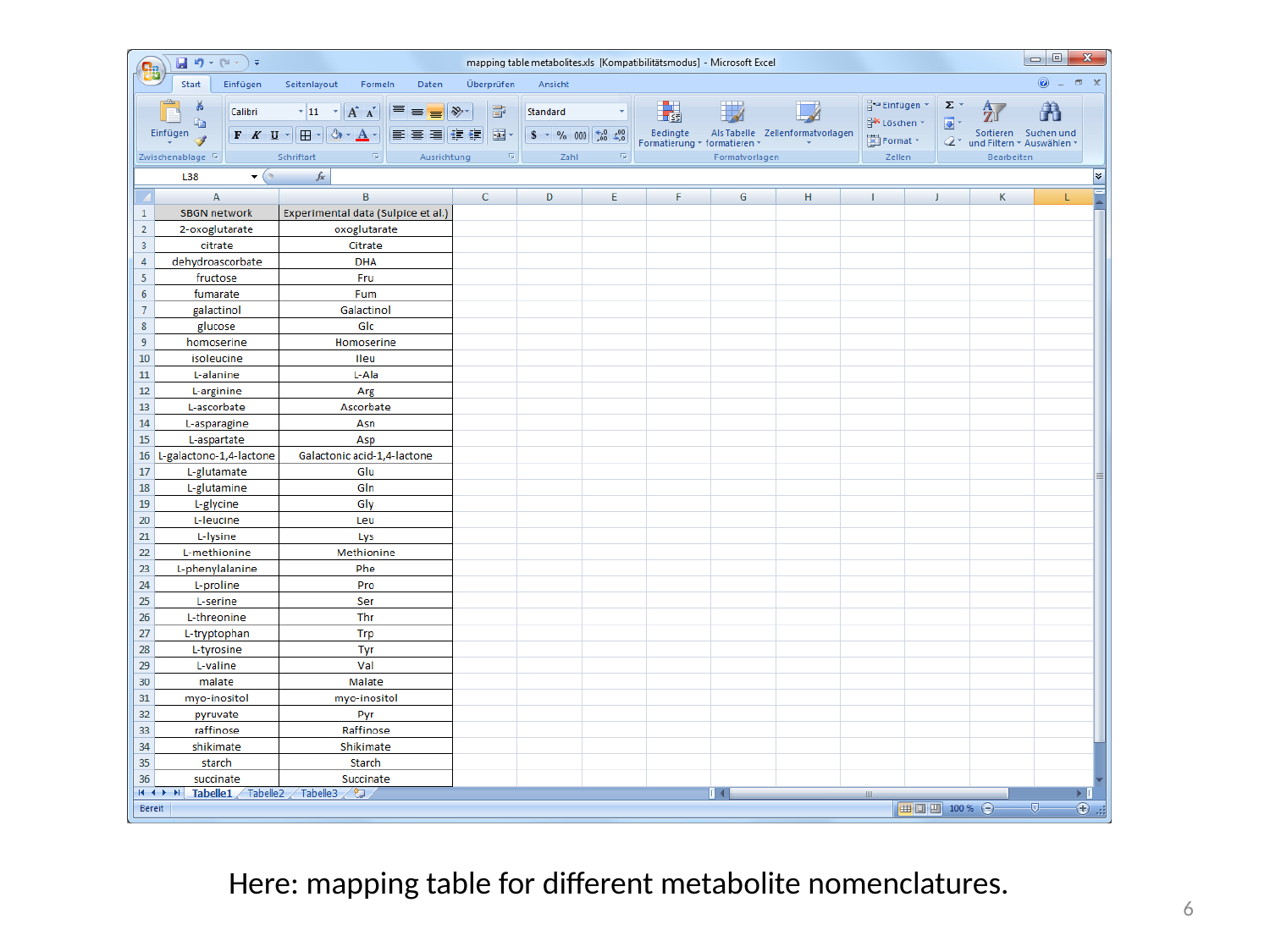

Here: mapping table for different metabolite nomenclatures.
6

## Slide 7
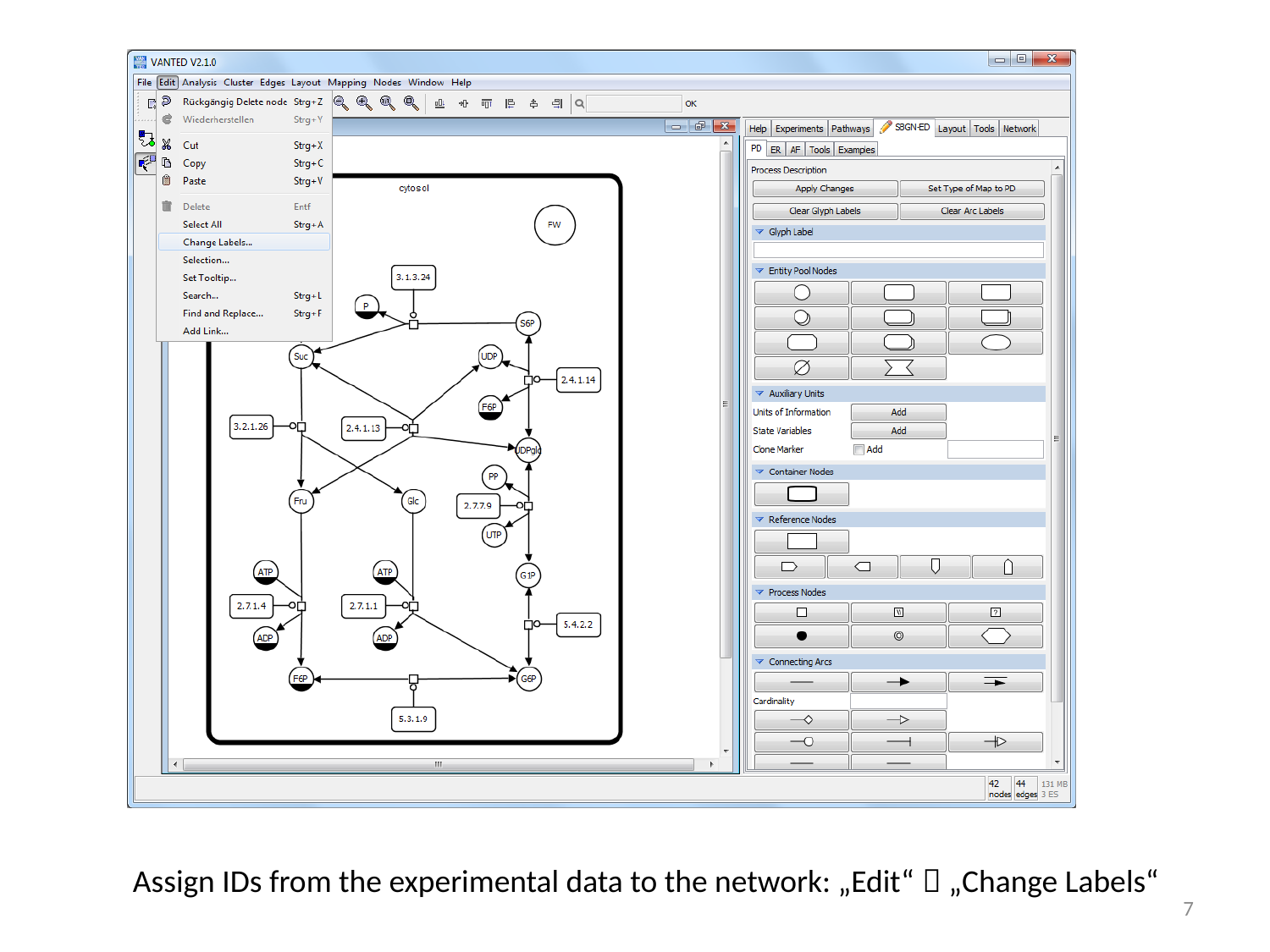

Assign IDs from the experimental data to the network: „Edit“  „Change Labels“
7

## Slide 8
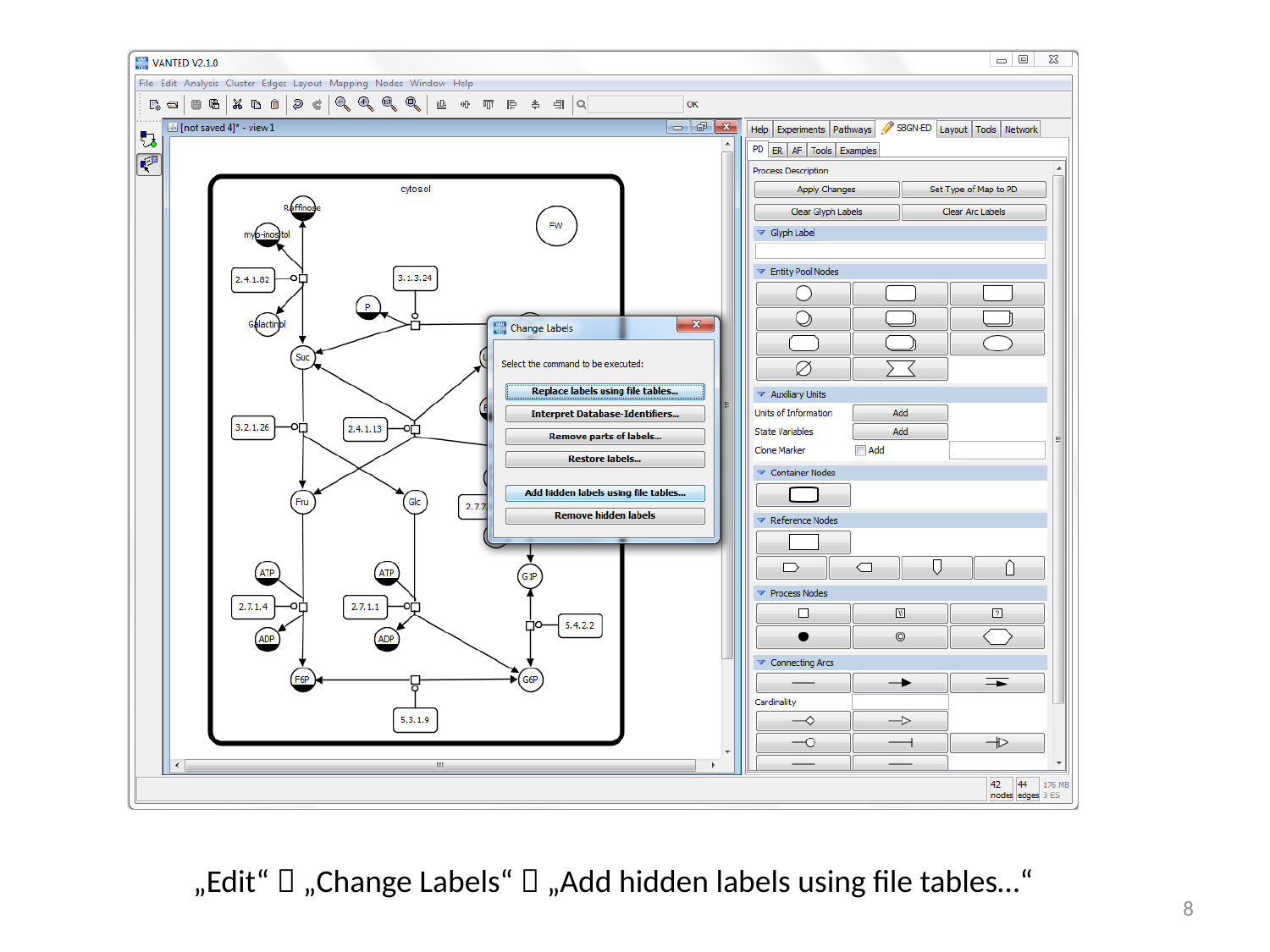

„Edit“  „Change Labels“  „Add hidden labels using file tables…“
8

## Slide 9
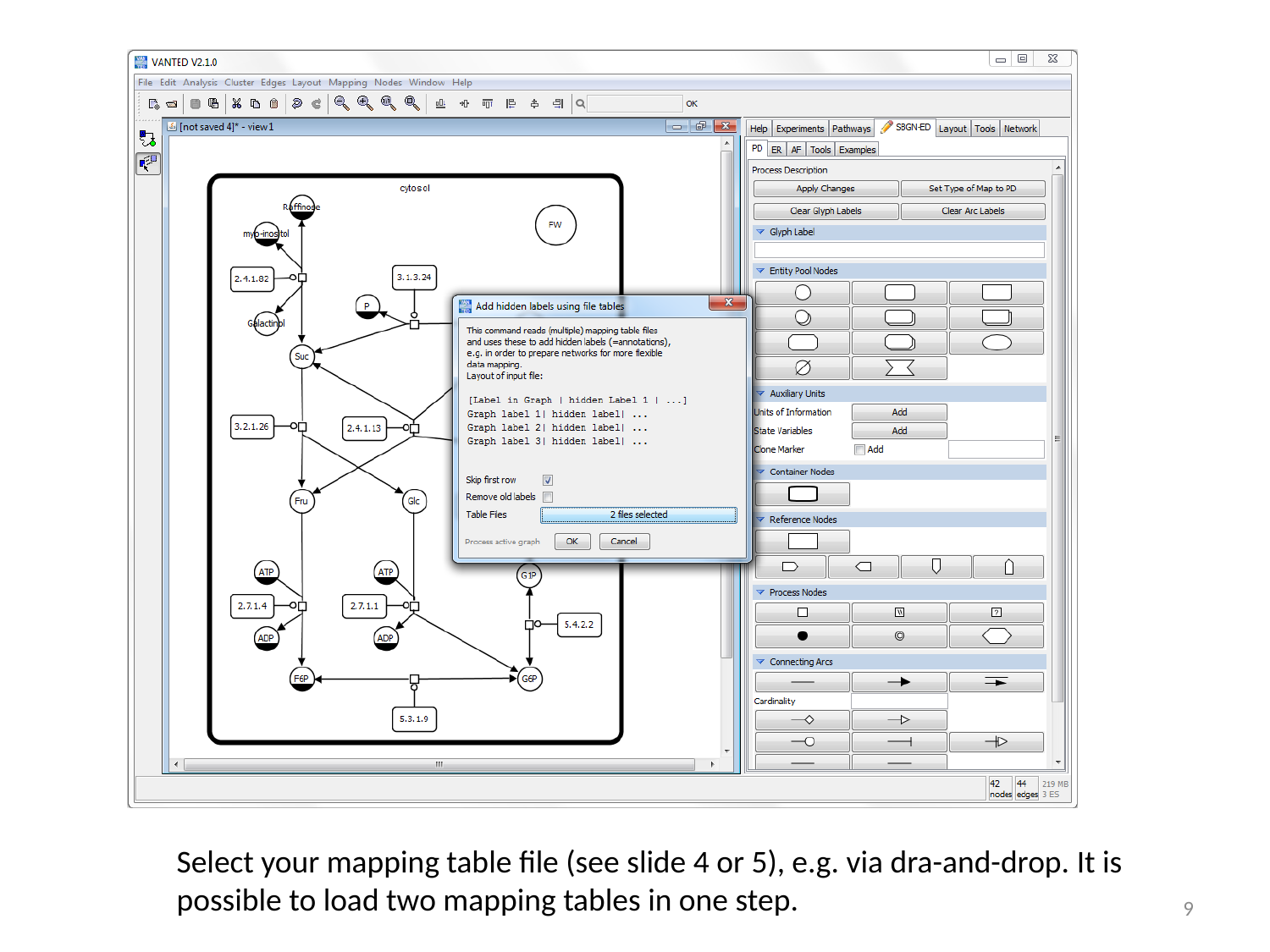

Select your mapping table file (see slide 4 or 5), e.g. via dra-and-drop. It is possible to load two mapping tables in one step.
9

## Slide 10
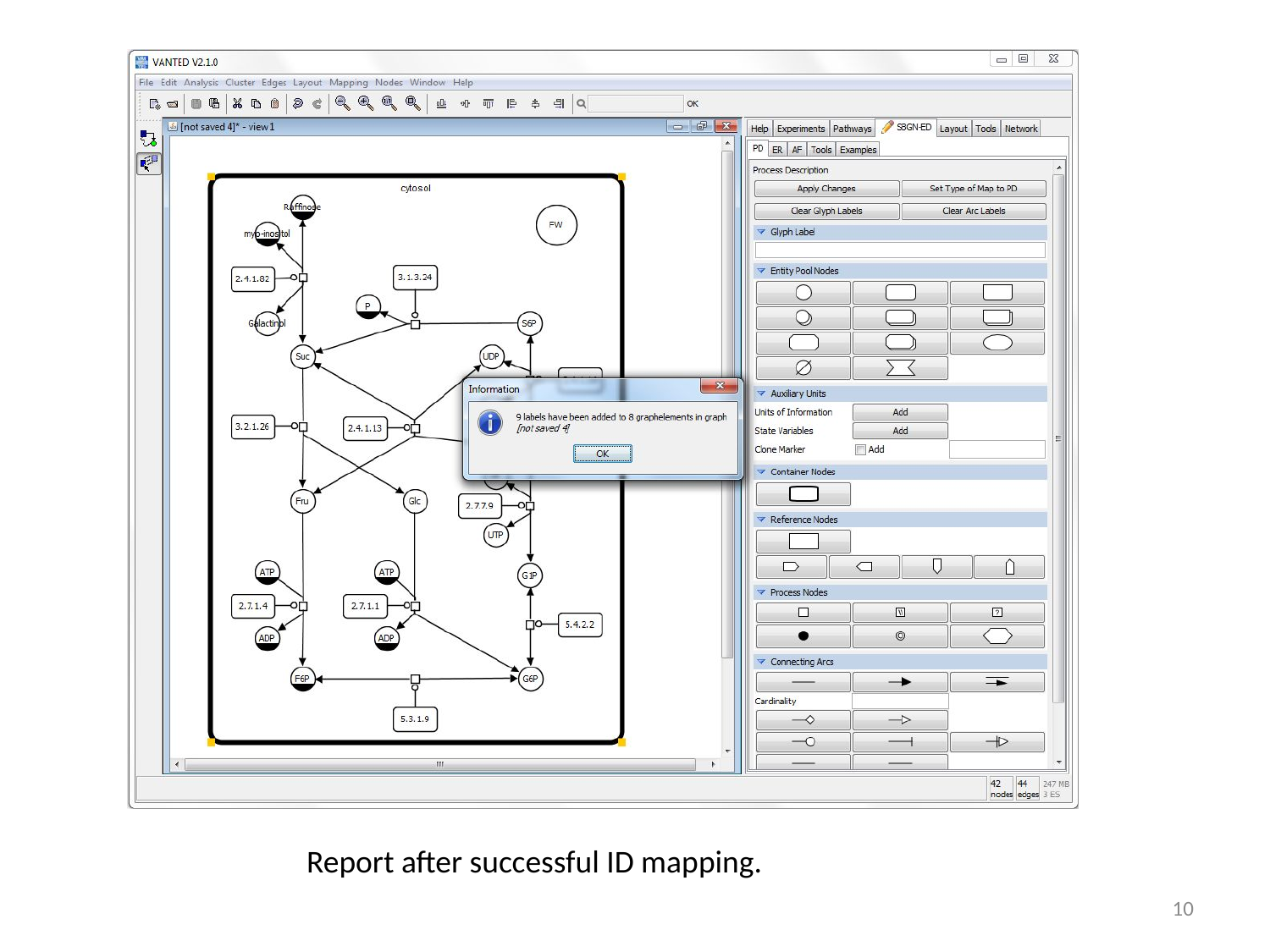

Report after successful ID mapping.
10

## Slide 11
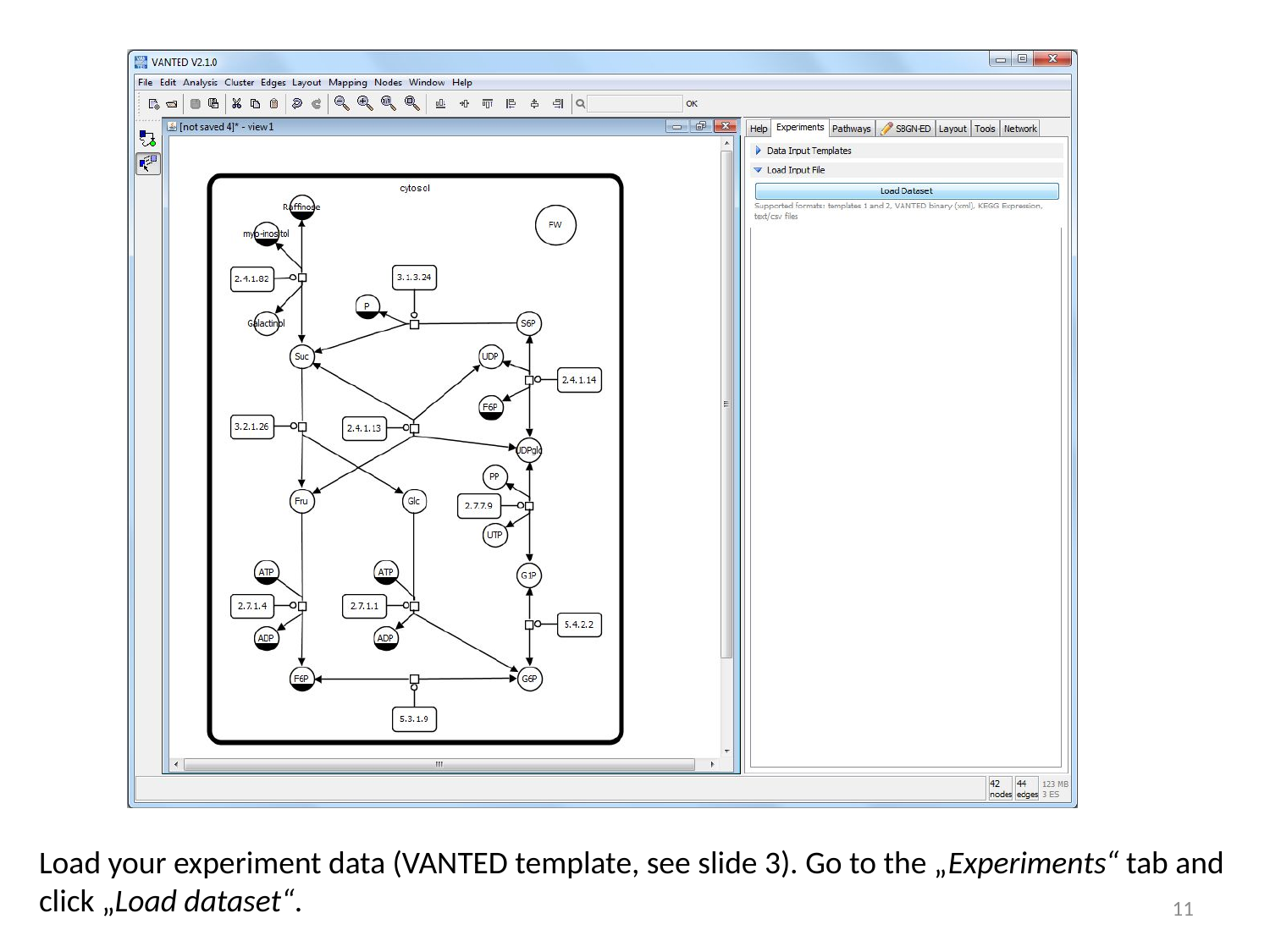

Load your experiment data (VANTED template, see slide 3). Go to the „Experiments“ tab and click „Load dataset“.
11

## Slide 12
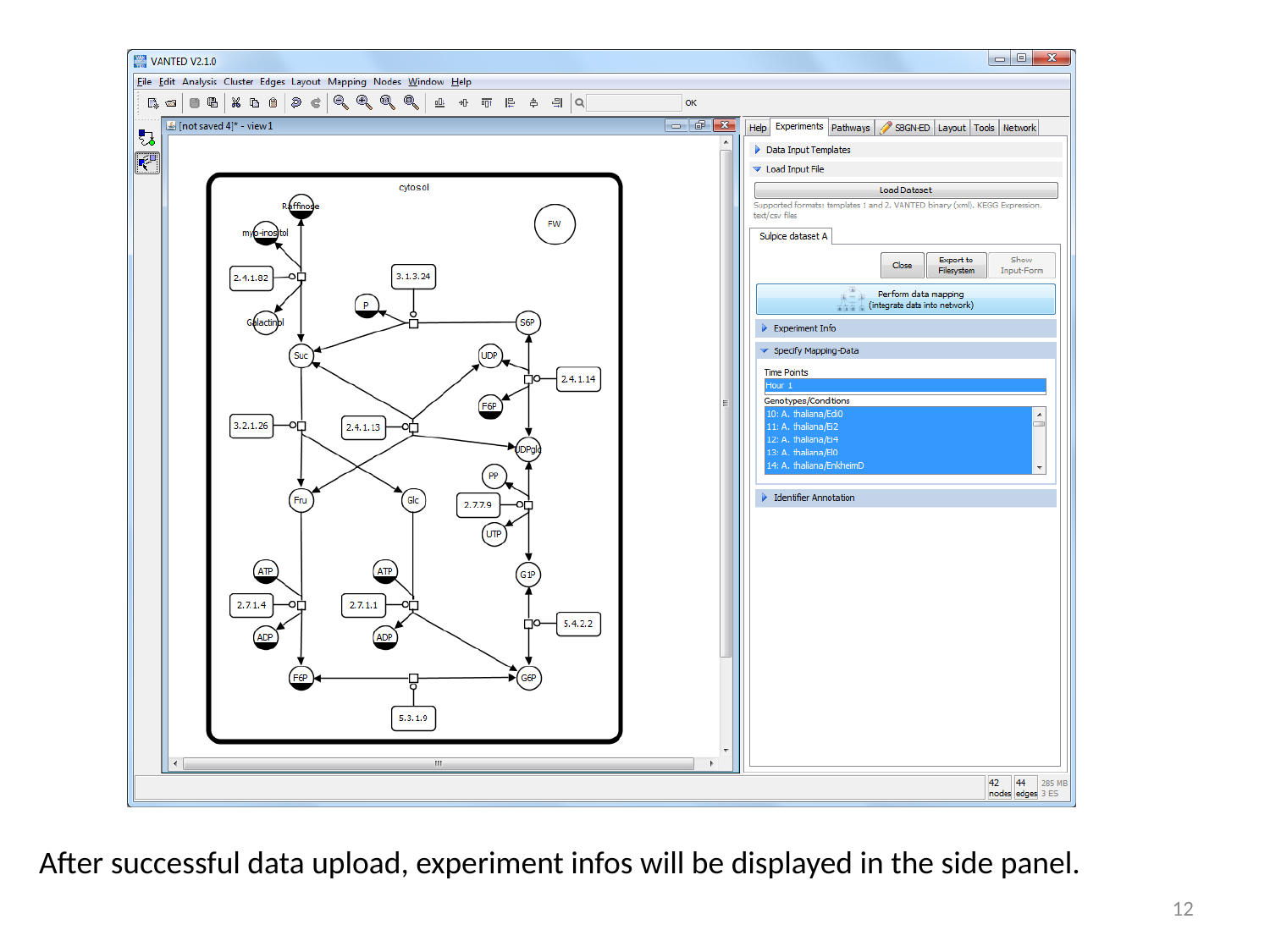

After successful data upload, experiment infos will be displayed in the side panel.
12

## Slide 13
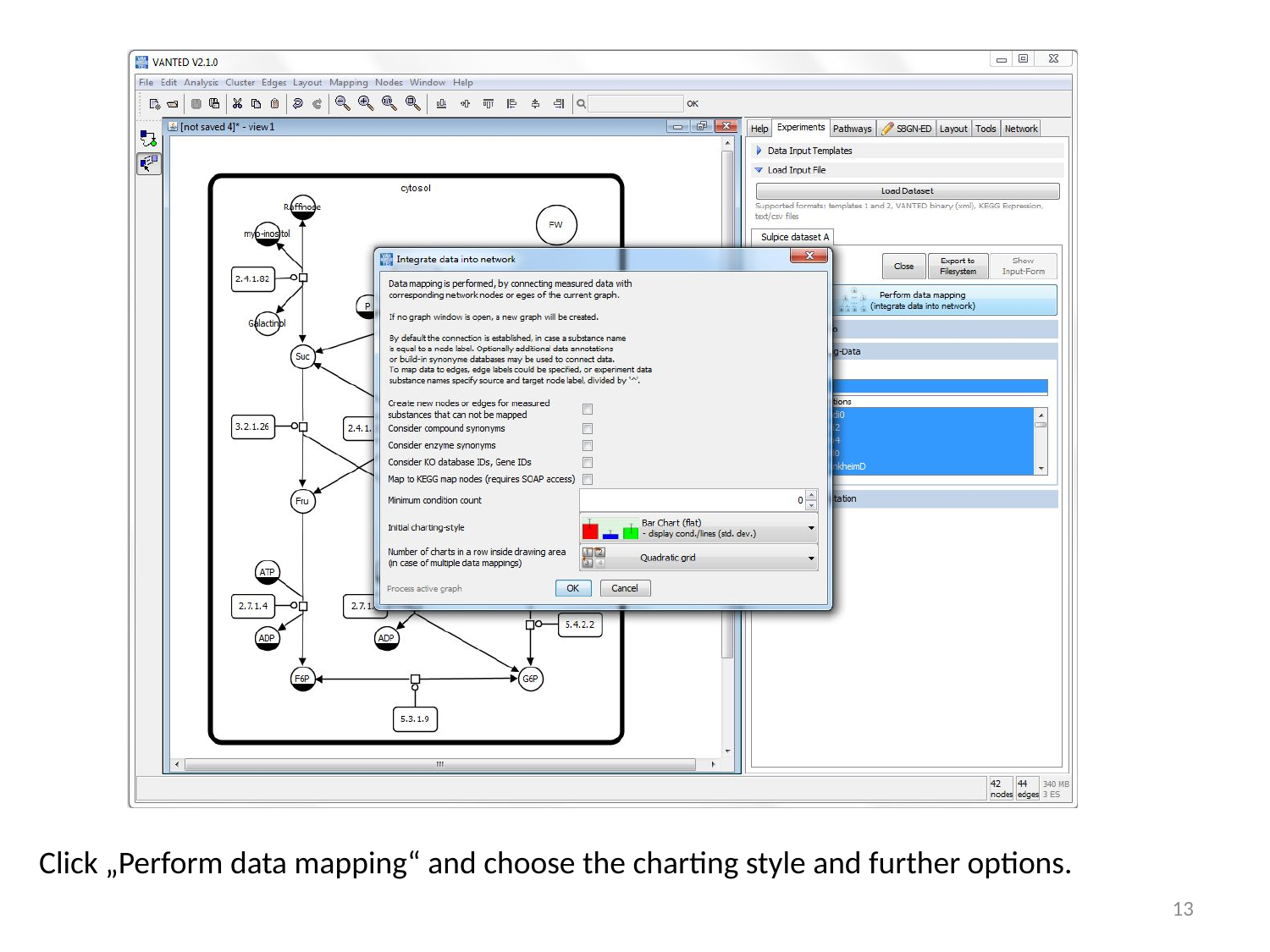

Click „Perform data mapping“ and choose the charting style and further options.
13

## Slide 14
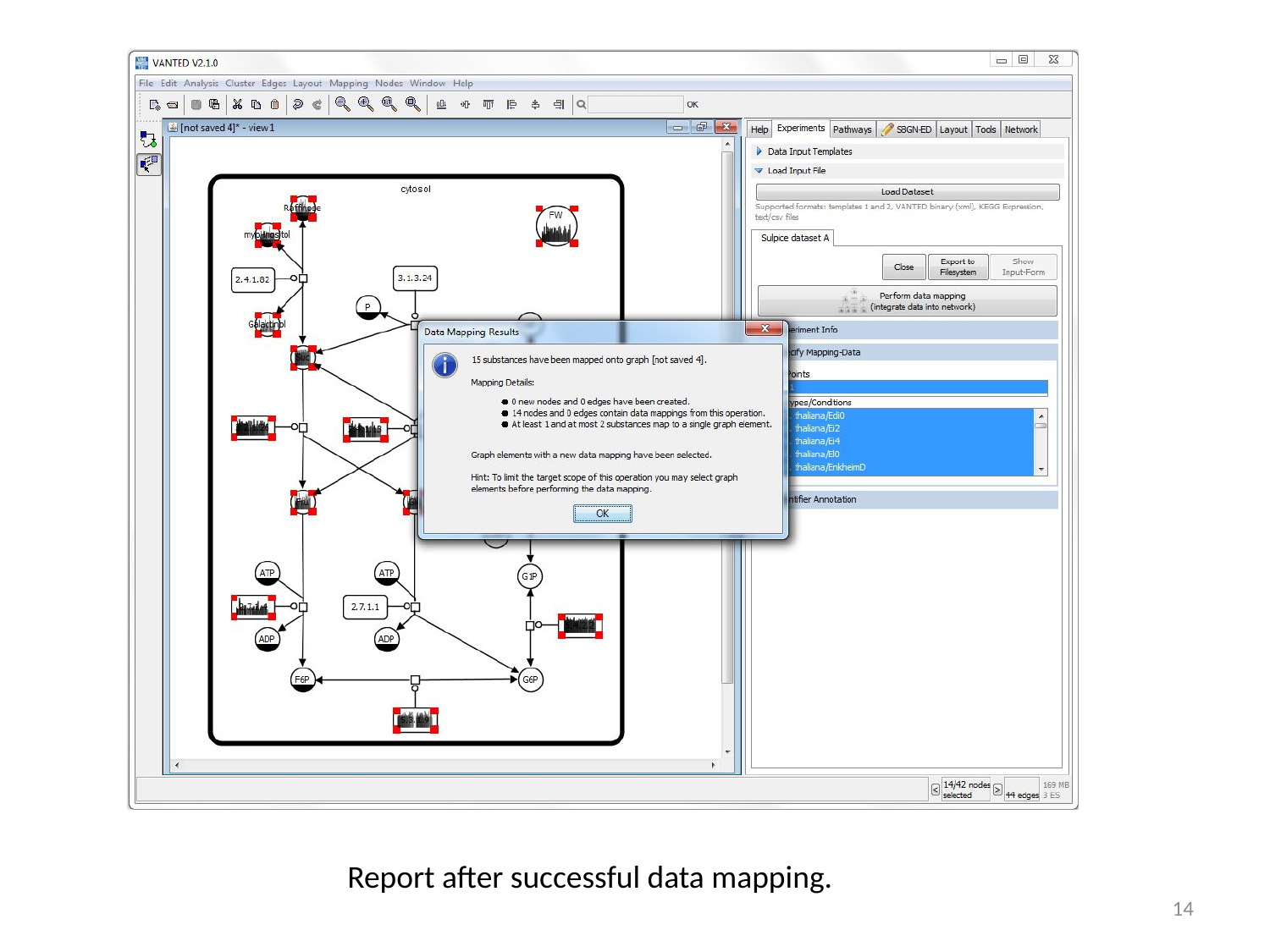

Report after successful data mapping.
14

## Slide 15
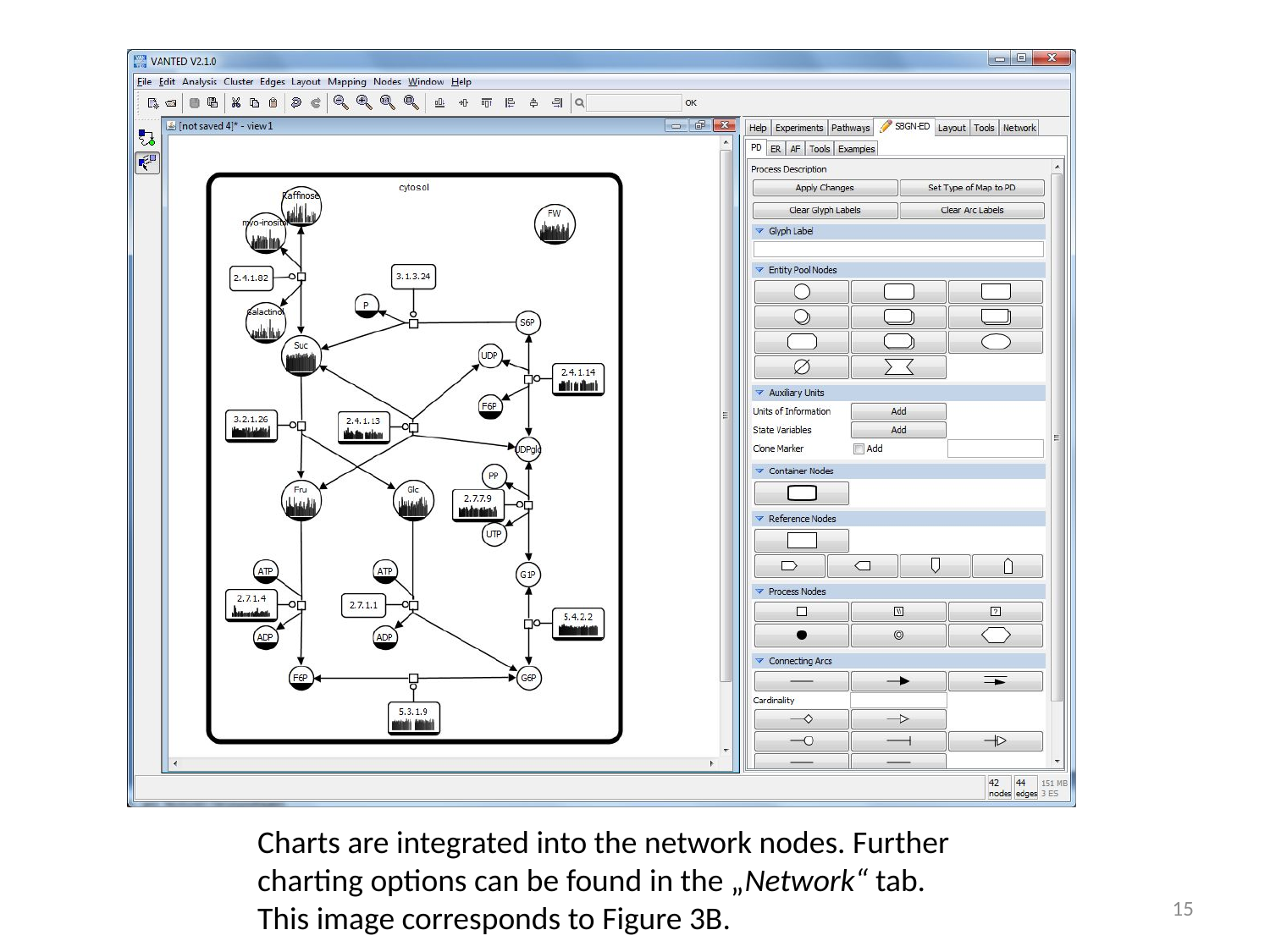

Charts are integrated into the network nodes. Further charting options can be found in the „Network“ tab.
This image corresponds to Figure 3B.
15

## Slide 16
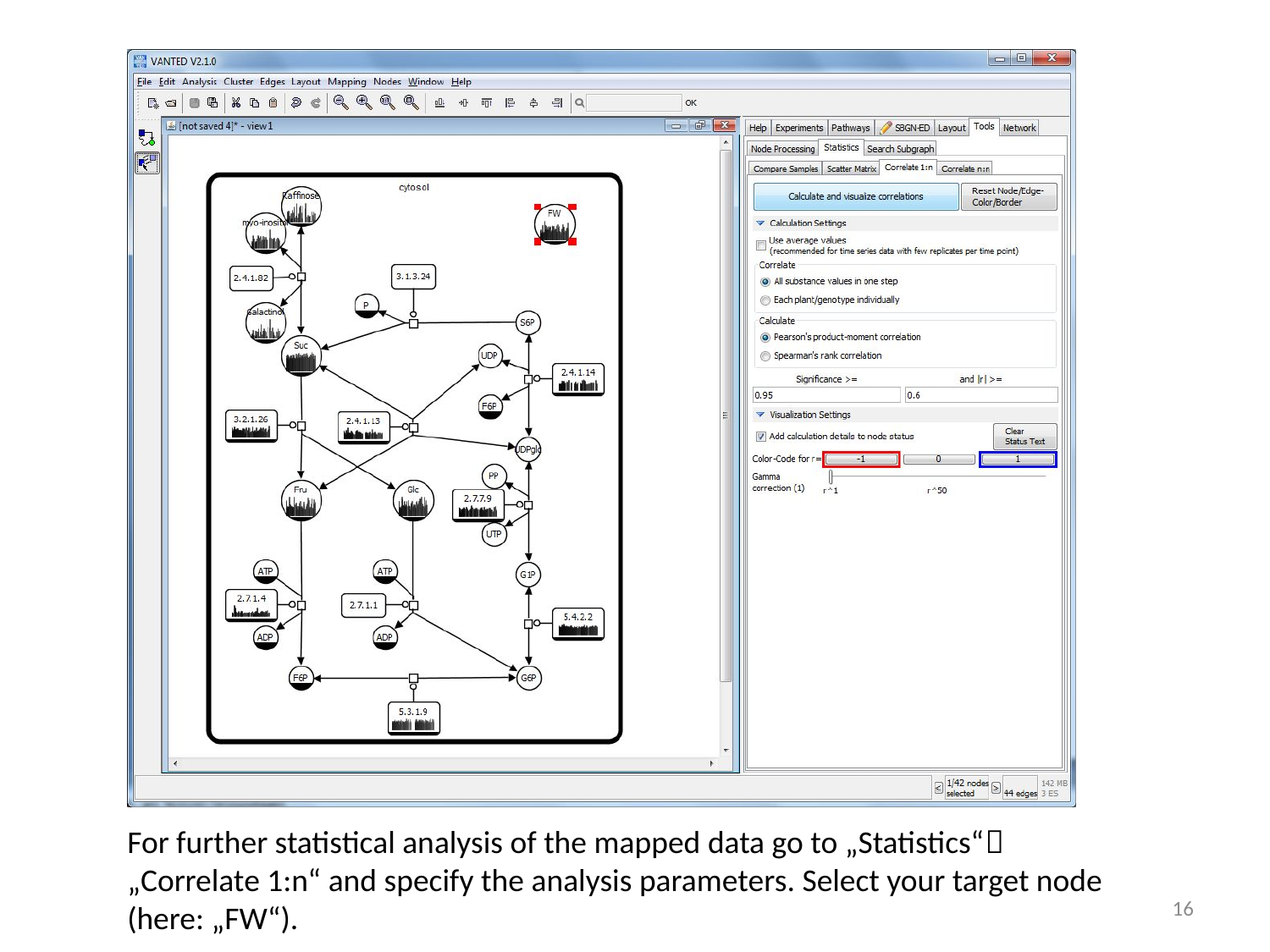

For further statistical analysis of the mapped data go to „Statistics“ „Correlate 1:n“ and specify the analysis parameters. Select your target node (here: „FW“).
16

## Slide 17
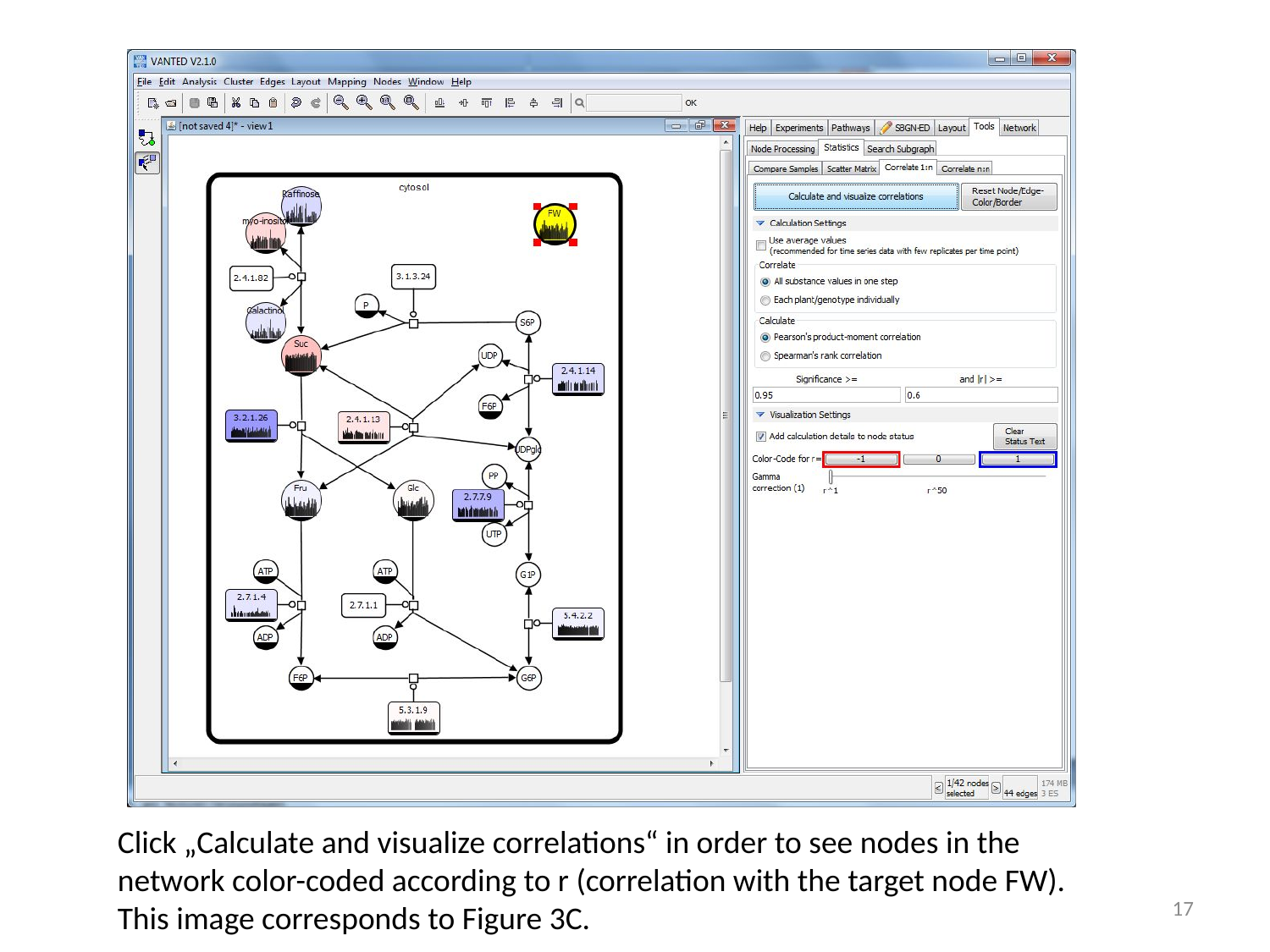

Click „Calculate and visualize correlations“ in order to see nodes in the network color-coded according to r (correlation with the target node FW).
This image corresponds to Figure 3C.
17

## Slide 18
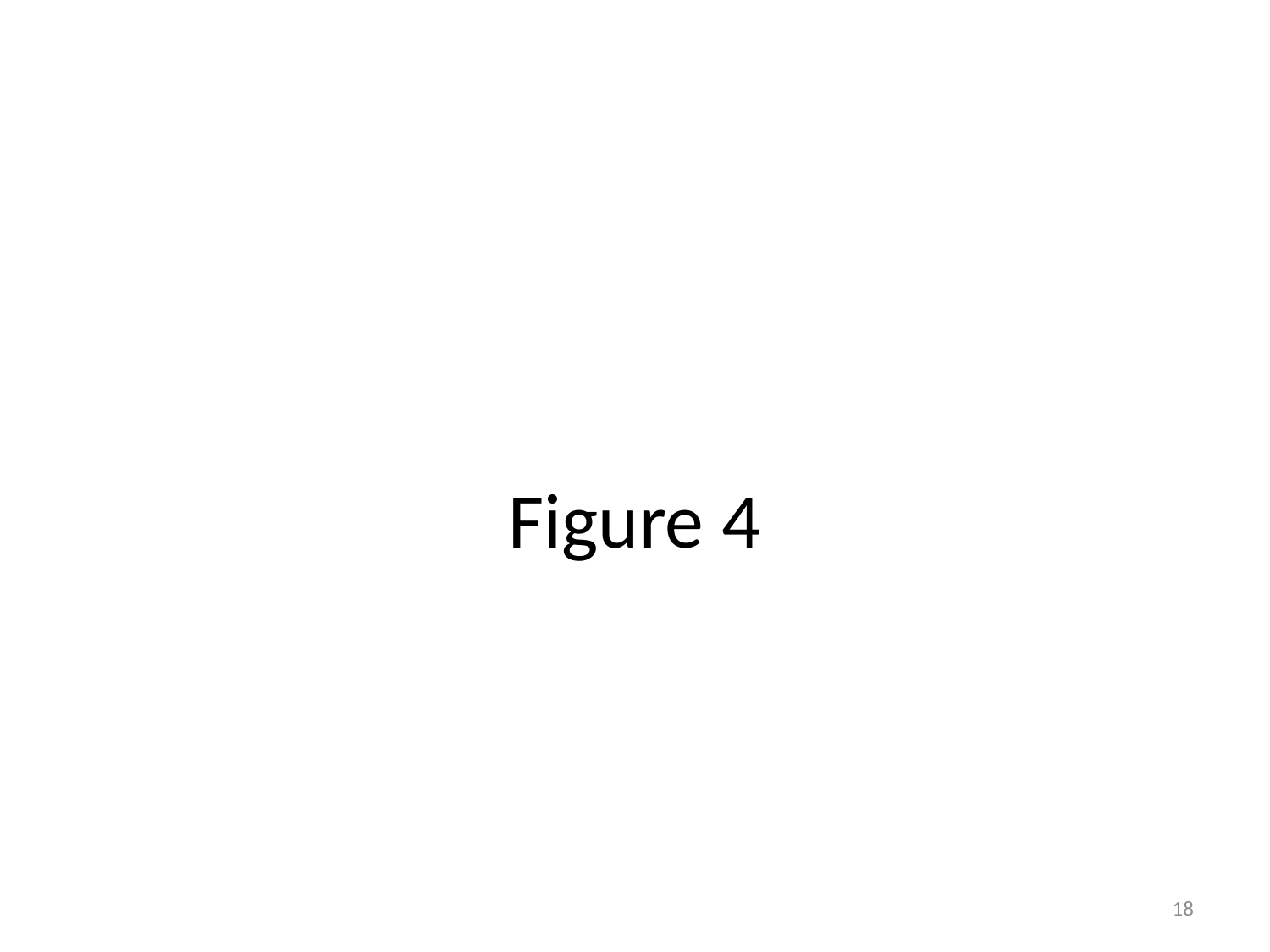

# Figure 4
18

## Slide 19
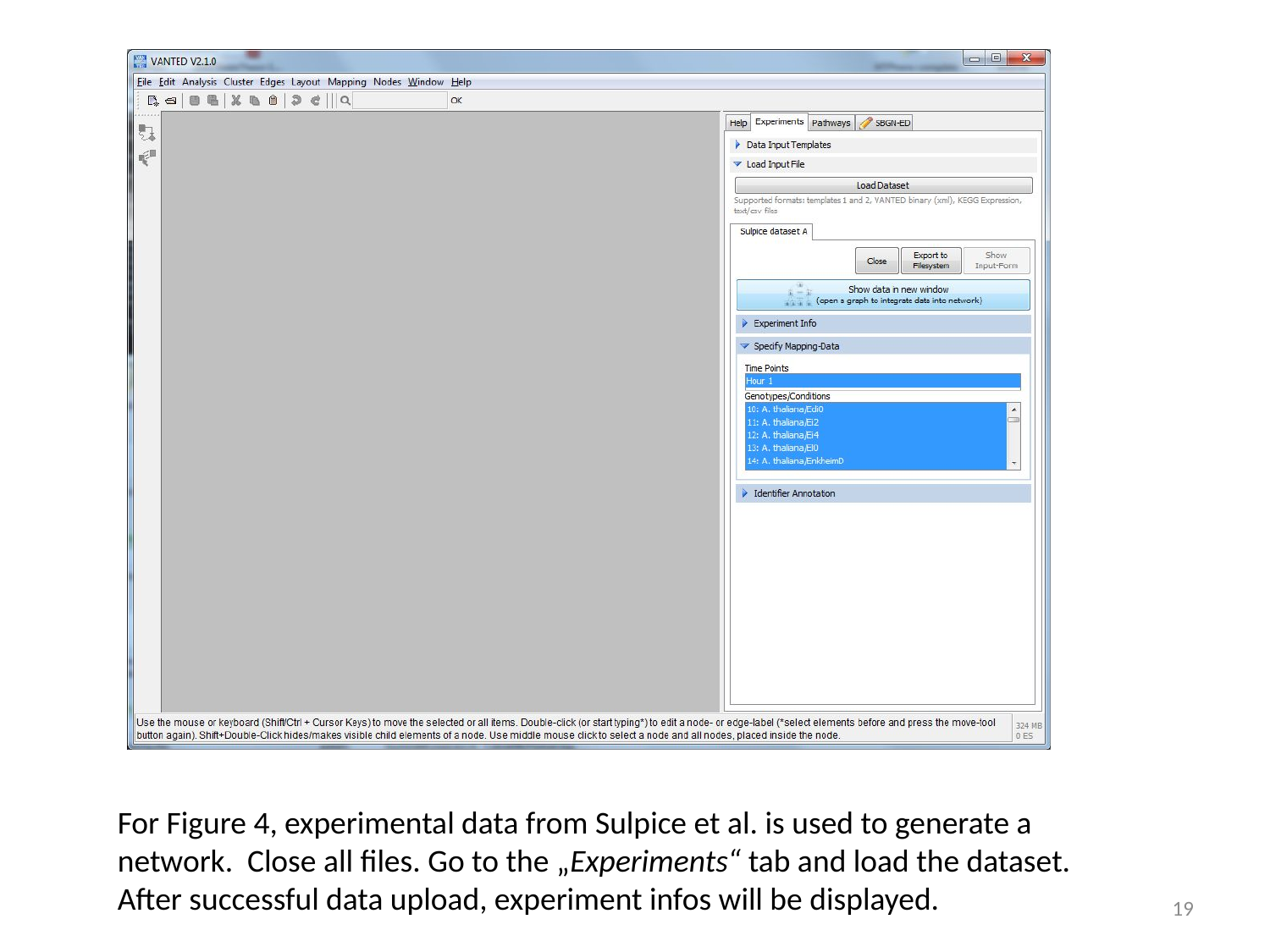

For Figure 4, experimental data from Sulpice et al. is used to generate a network. Close all files. Go to the „Experiments“ tab and load the dataset. After successful data upload, experiment infos will be displayed.
19

## Slide 20
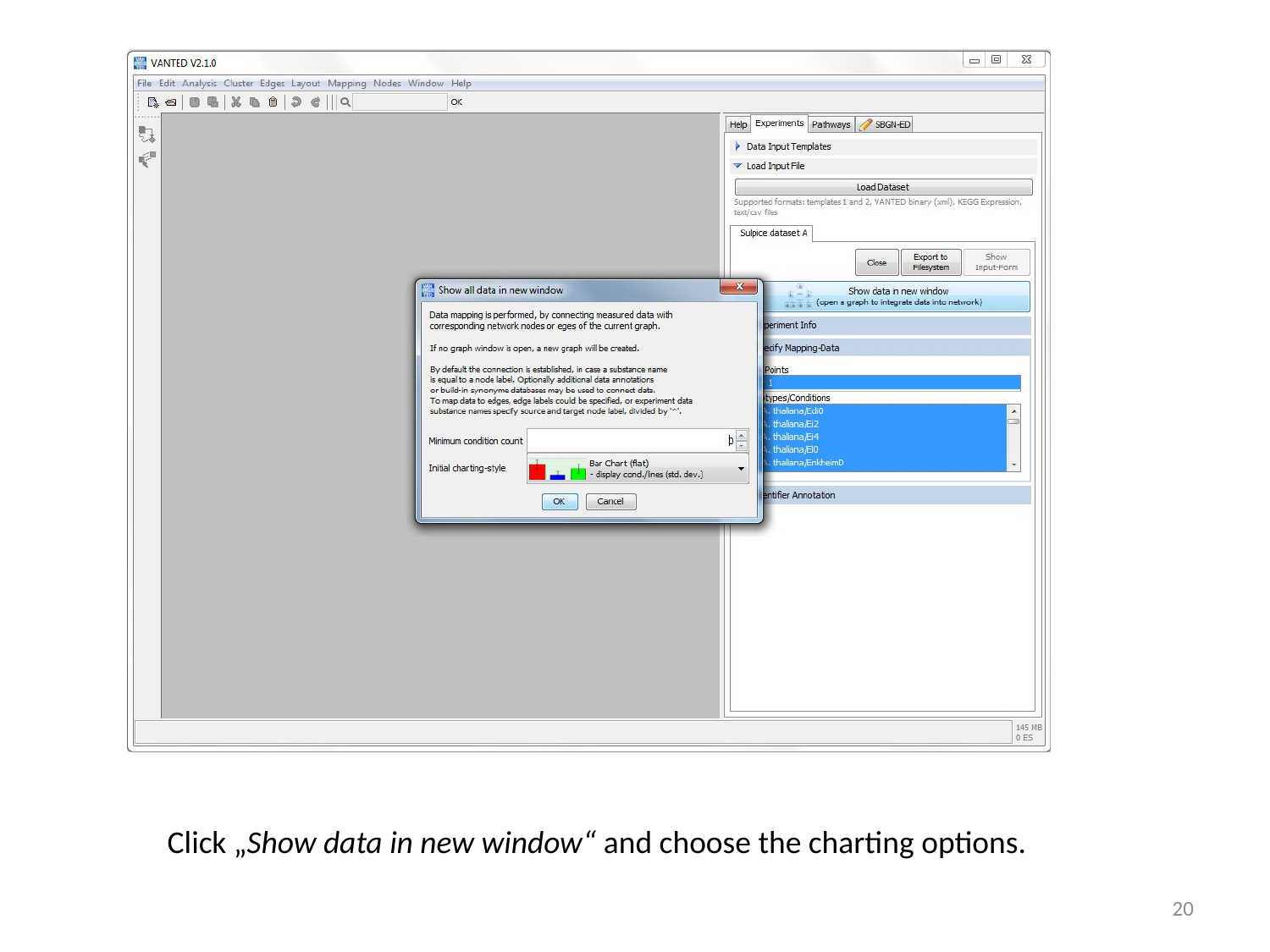

Click „Show data in new window“ and choose the charting options.
20

## Slide 21
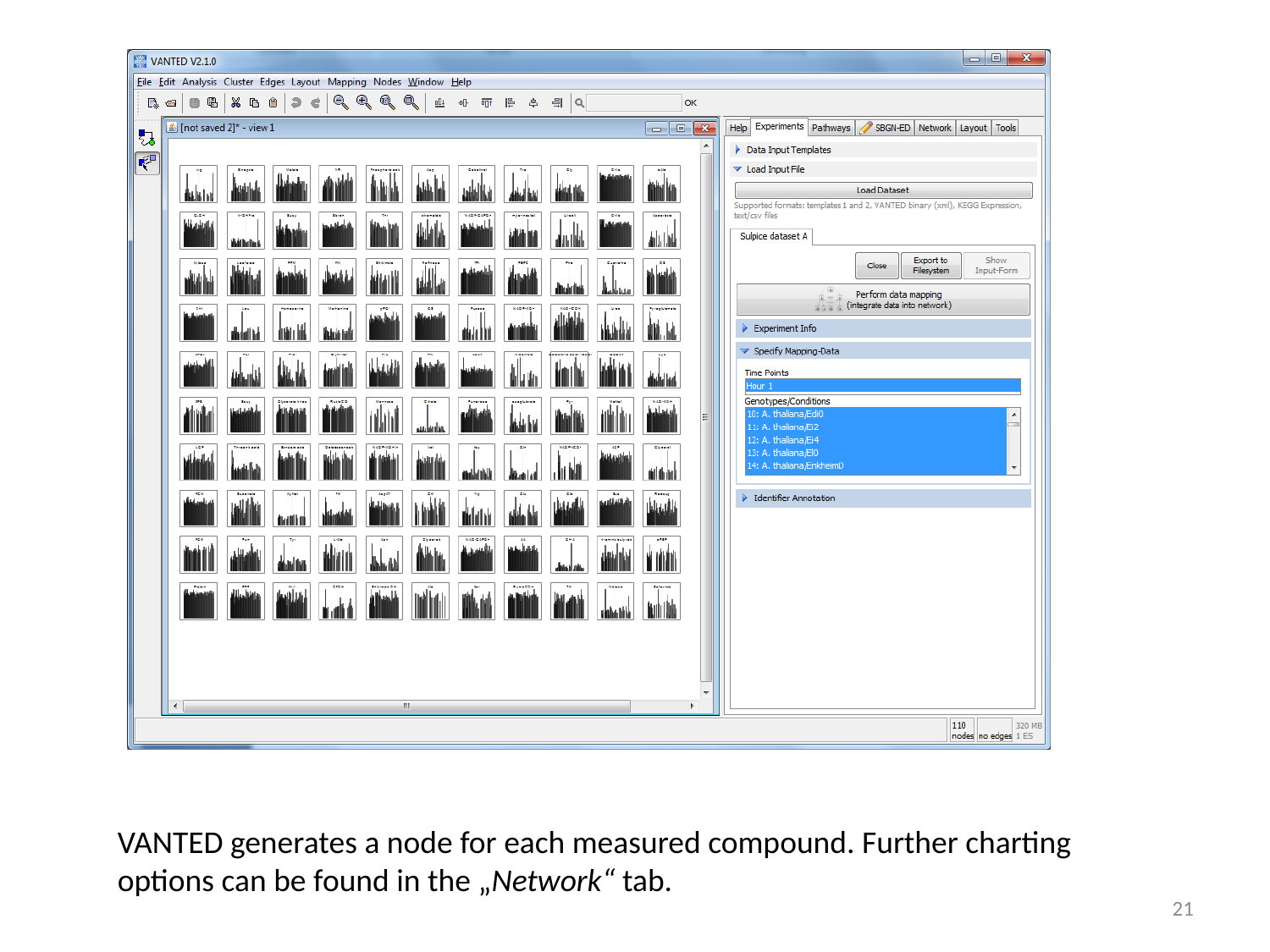

VANTED generates a node for each measured compound. Further charting options can be found in the „Network“ tab.
21

## Slide 22
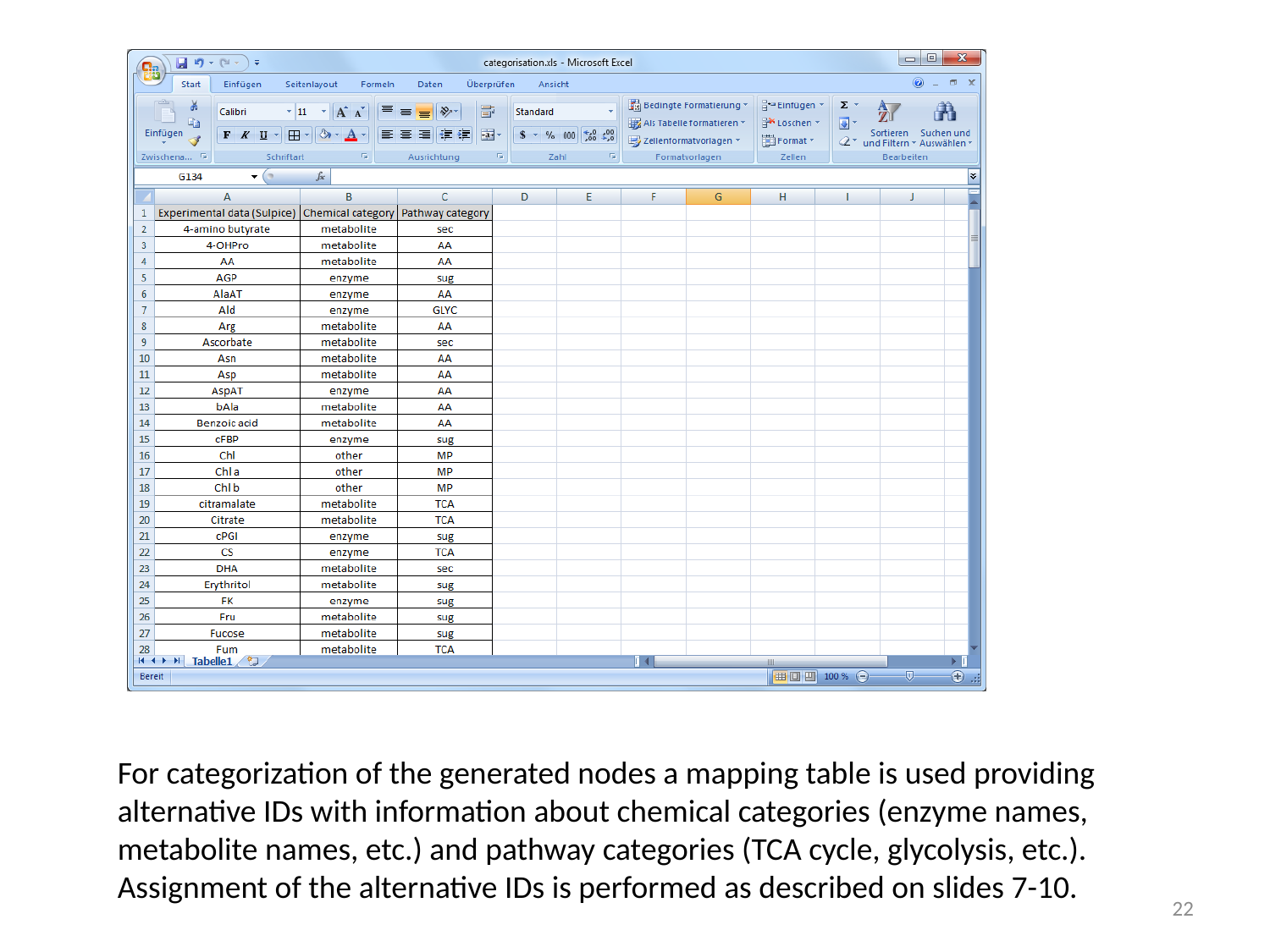

For categorization of the generated nodes a mapping table is used providing alternative IDs with information about chemical categories (enzyme names, metabolite names, etc.) and pathway categories (TCA cycle, glycolysis, etc.). Assignment of the alternative IDs is performed as described on slides 7-10.
22

## Slide 23
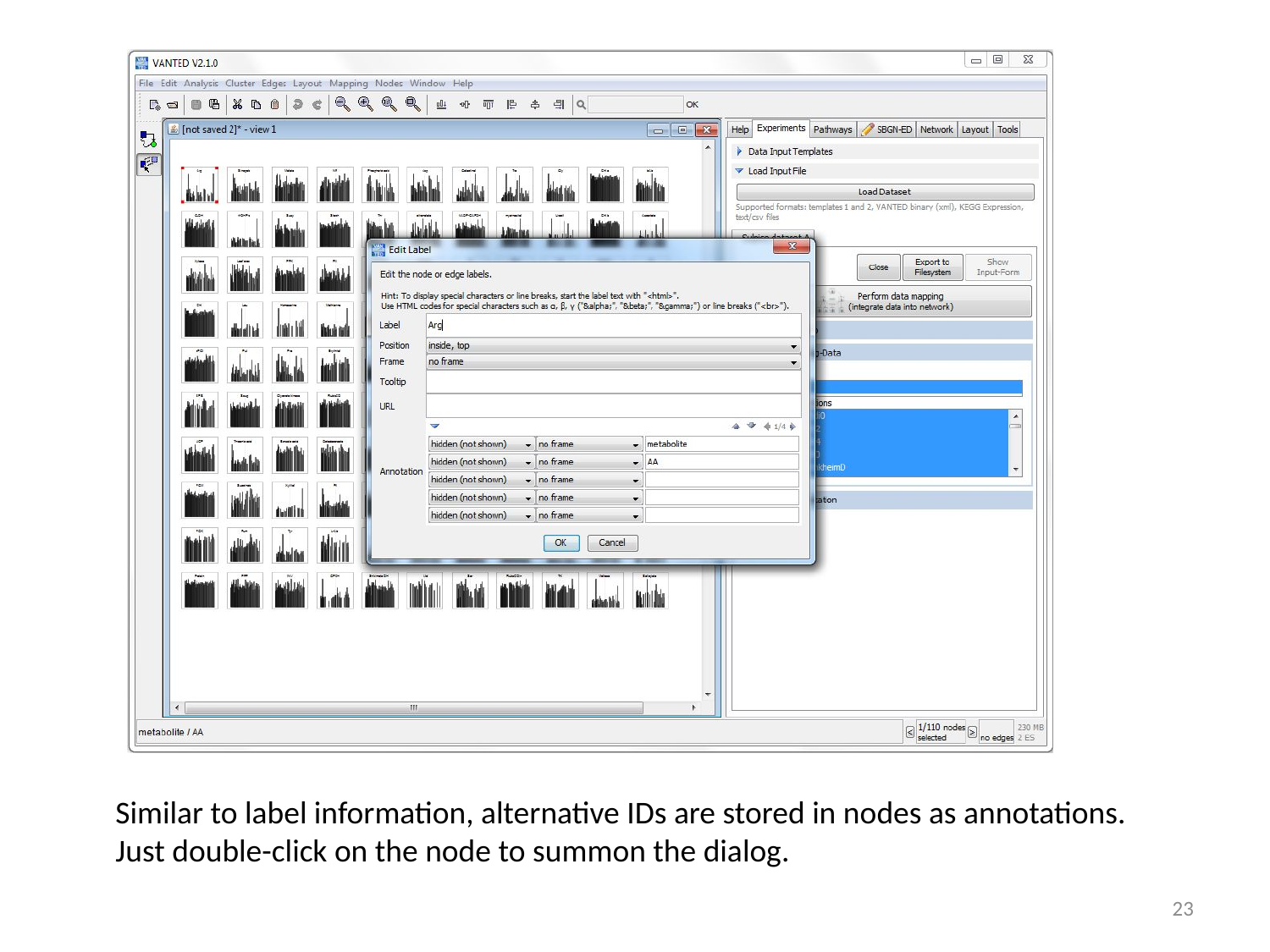

Similar to label information, alternative IDs are stored in nodes as annotations. Just double-click on the node to summon the dialog.
23

## Slide 24
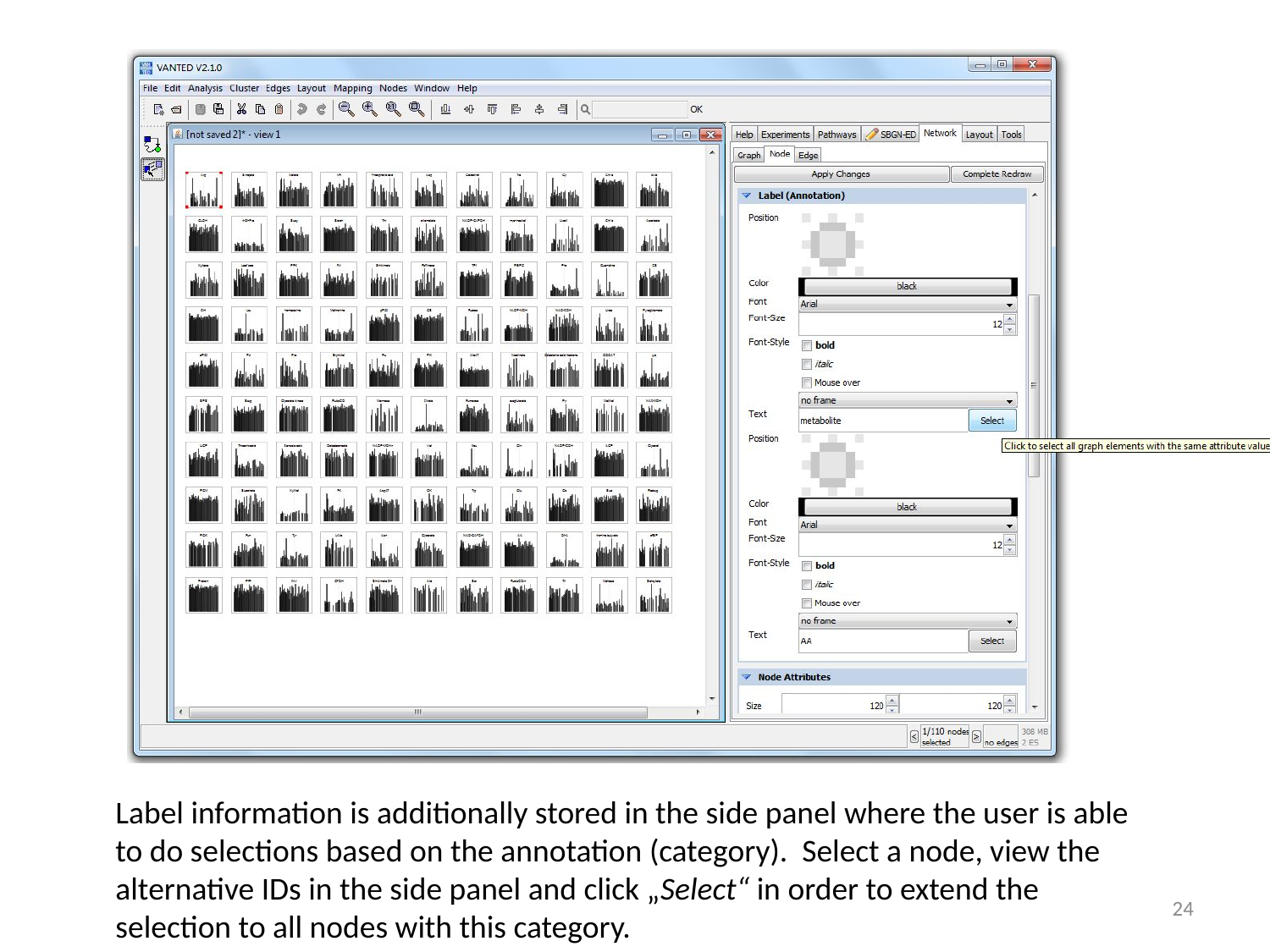

Label information is additionally stored in the side panel where the user is able to do selections based on the annotation (category). Select a node, view the alternative IDs in the side panel and click „Select“ in order to extend the selection to all nodes with this category.
24

## Slide 25
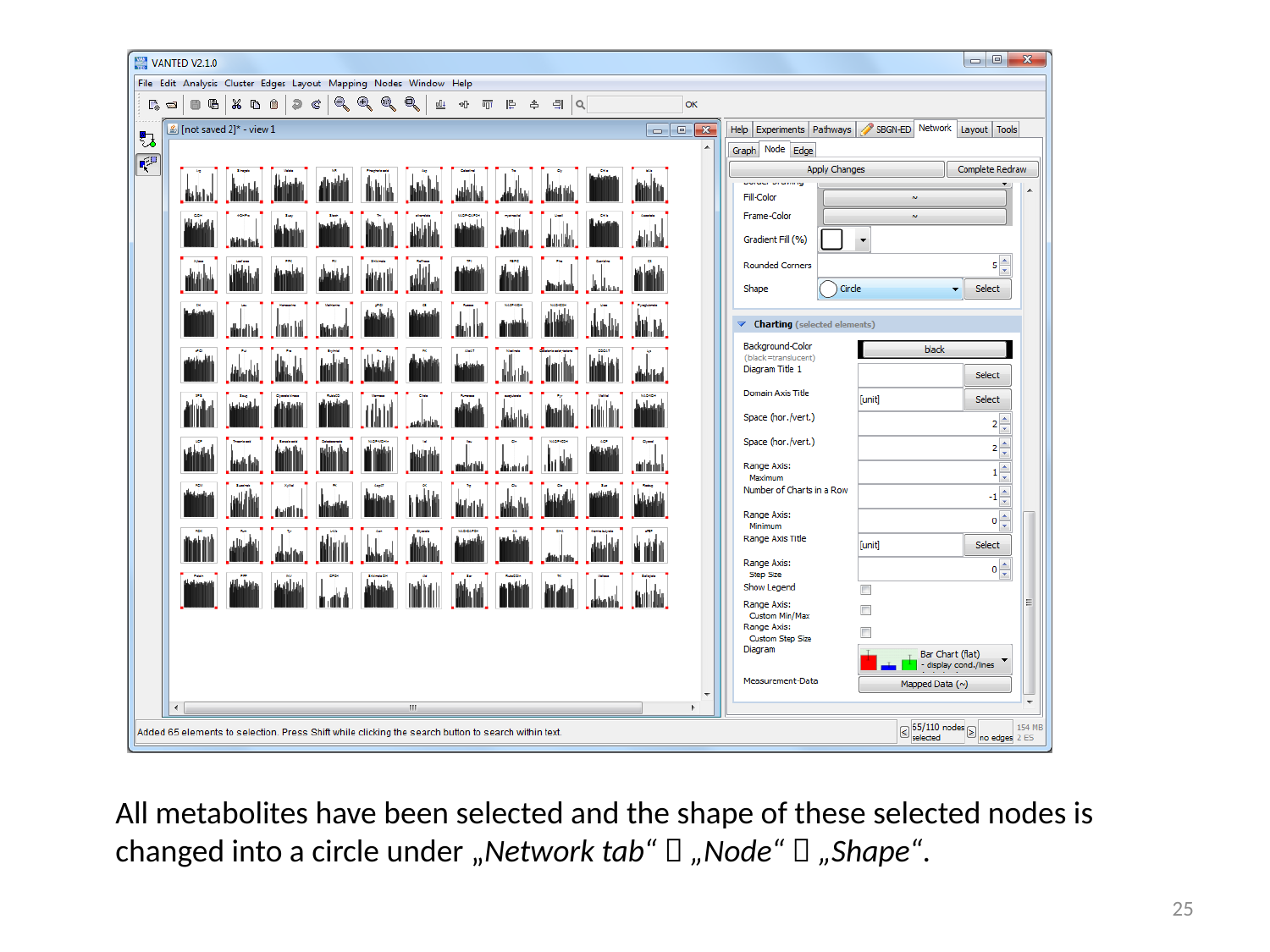

All metabolites have been selected and the shape of these selected nodes is changed into a circle under „Network tab“  „Node“  „Shape“.
25

## Slide 26
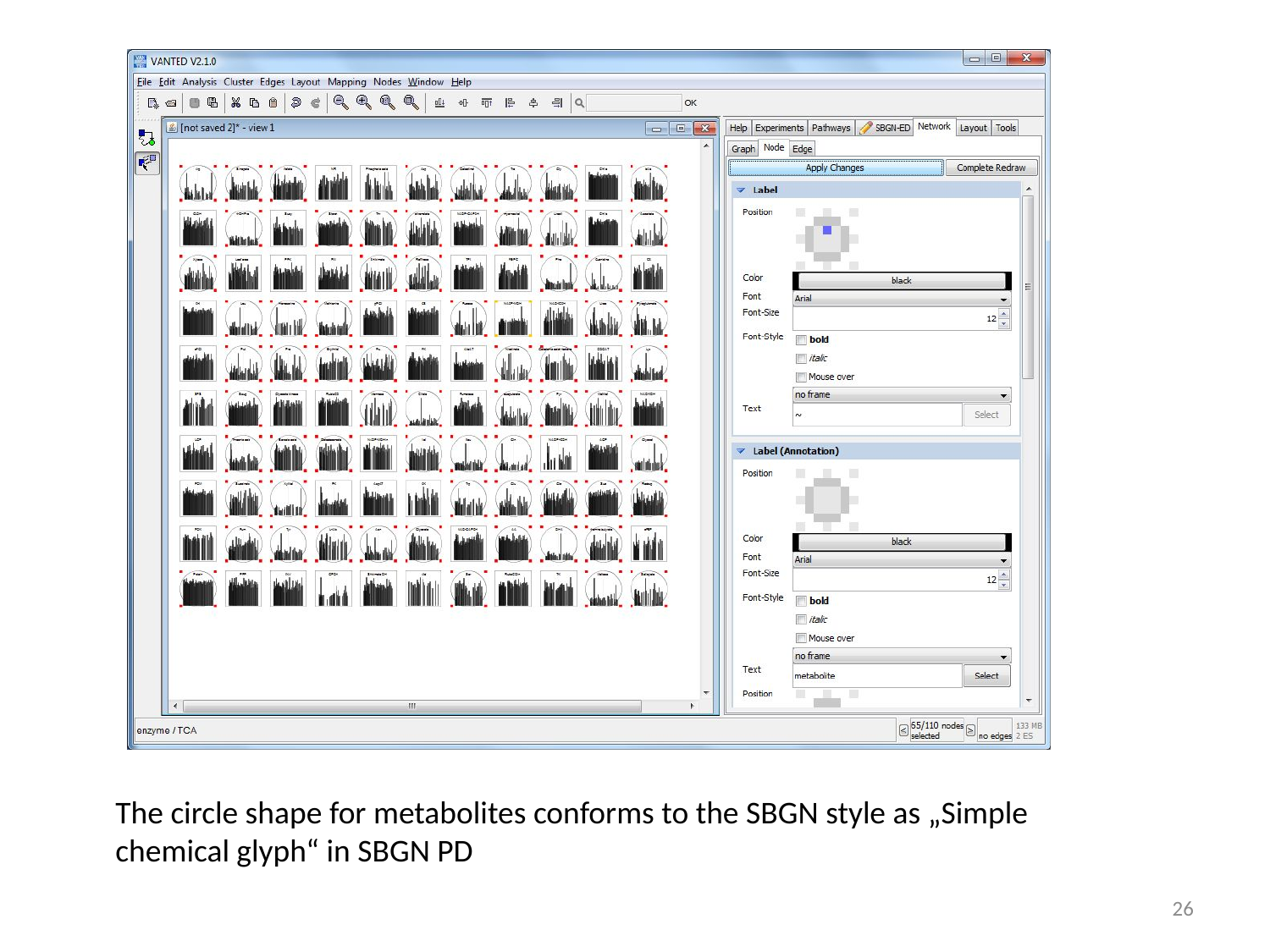

The circle shape for metabolites conforms to the SBGN style as „Simple chemical glyph“ in SBGN PD
26

## Slide 27
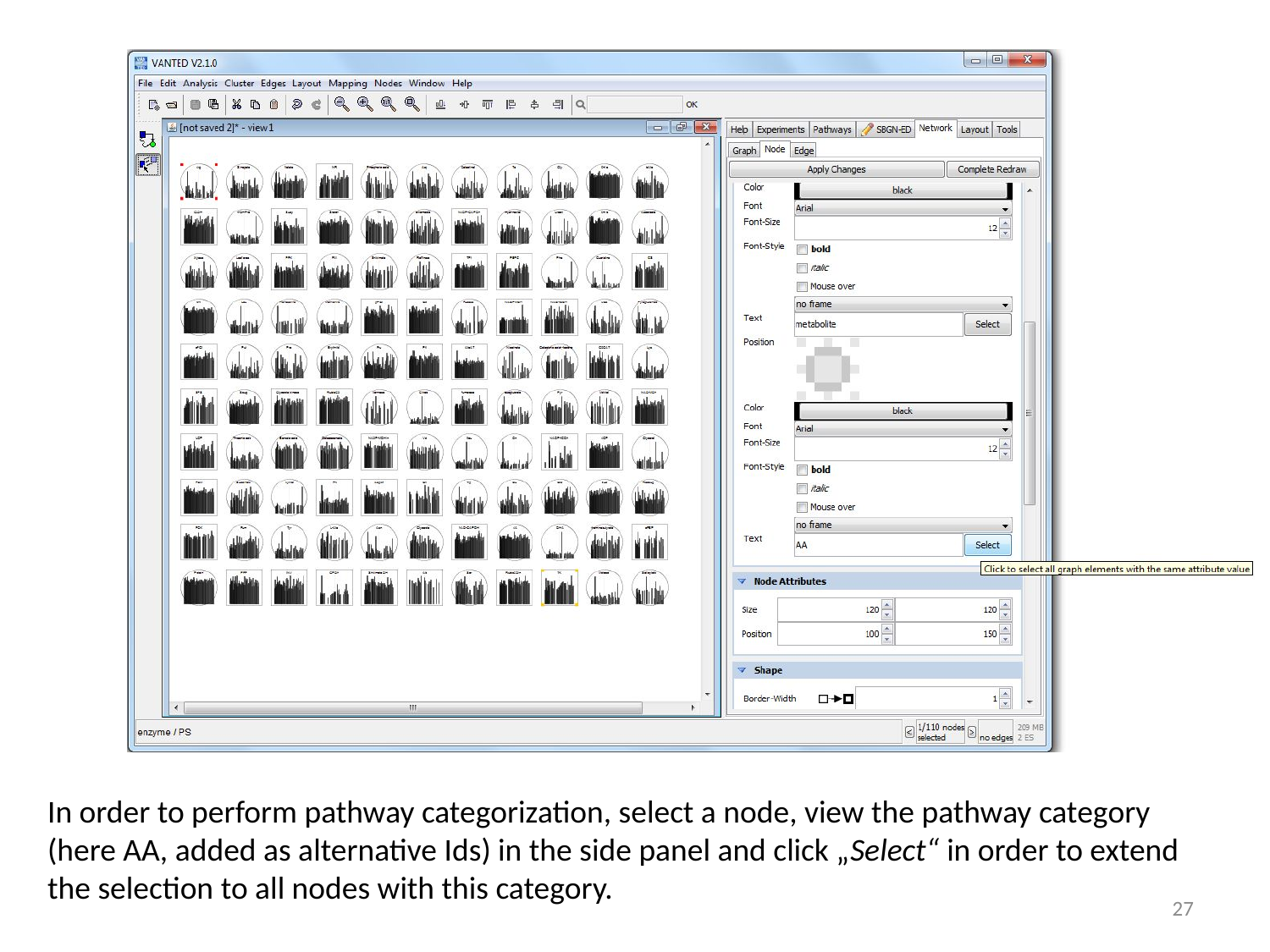

In order to perform pathway categorization, select a node, view the pathway category (here AA, added as alternative Ids) in the side panel and click „Select“ in order to extend the selection to all nodes with this category.
27

## Slide 28
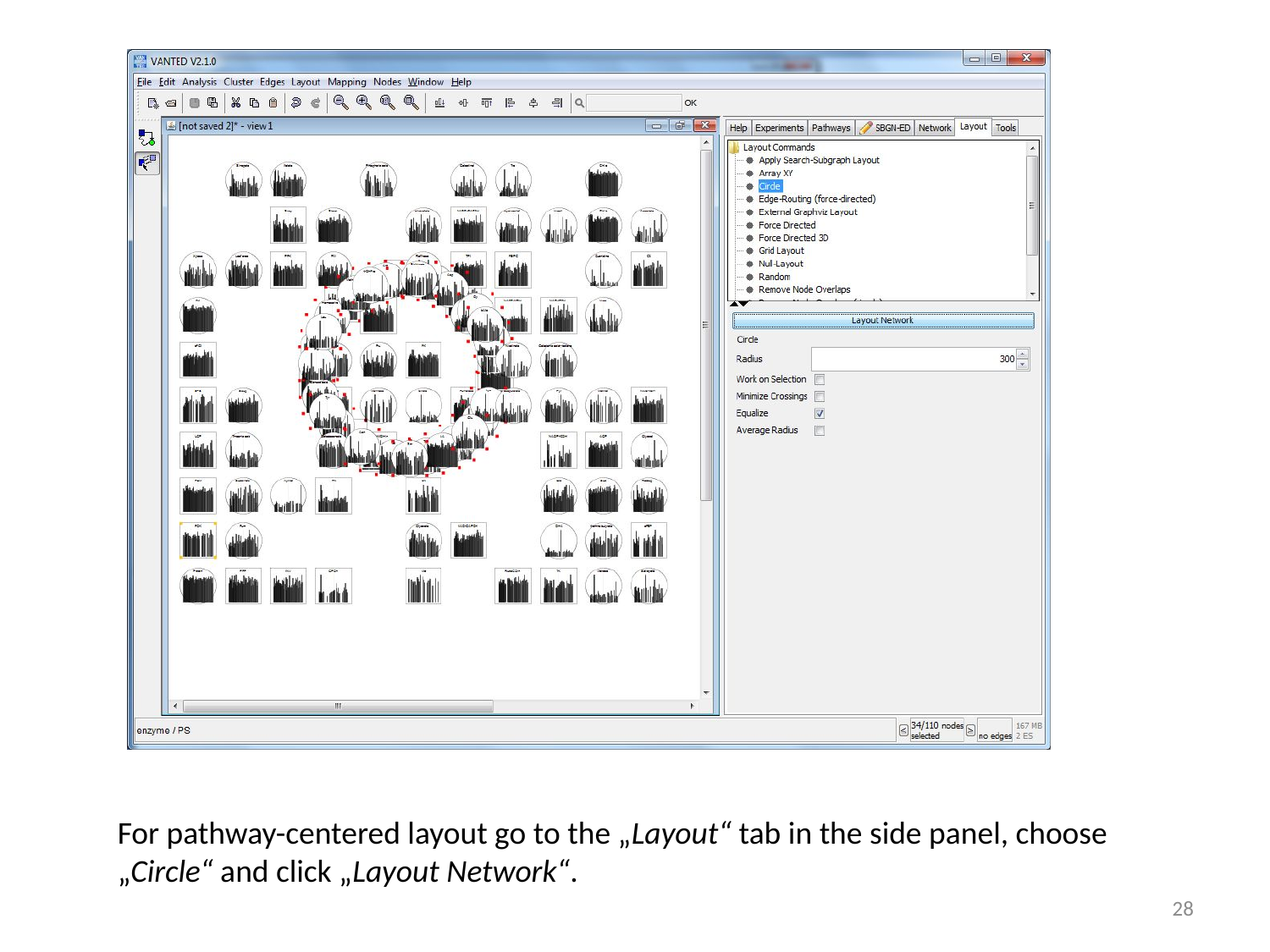

For pathway-centered layout go to the „Layout“ tab in the side panel, choose „Circle“ and click „Layout Network“.
28

## Slide 29
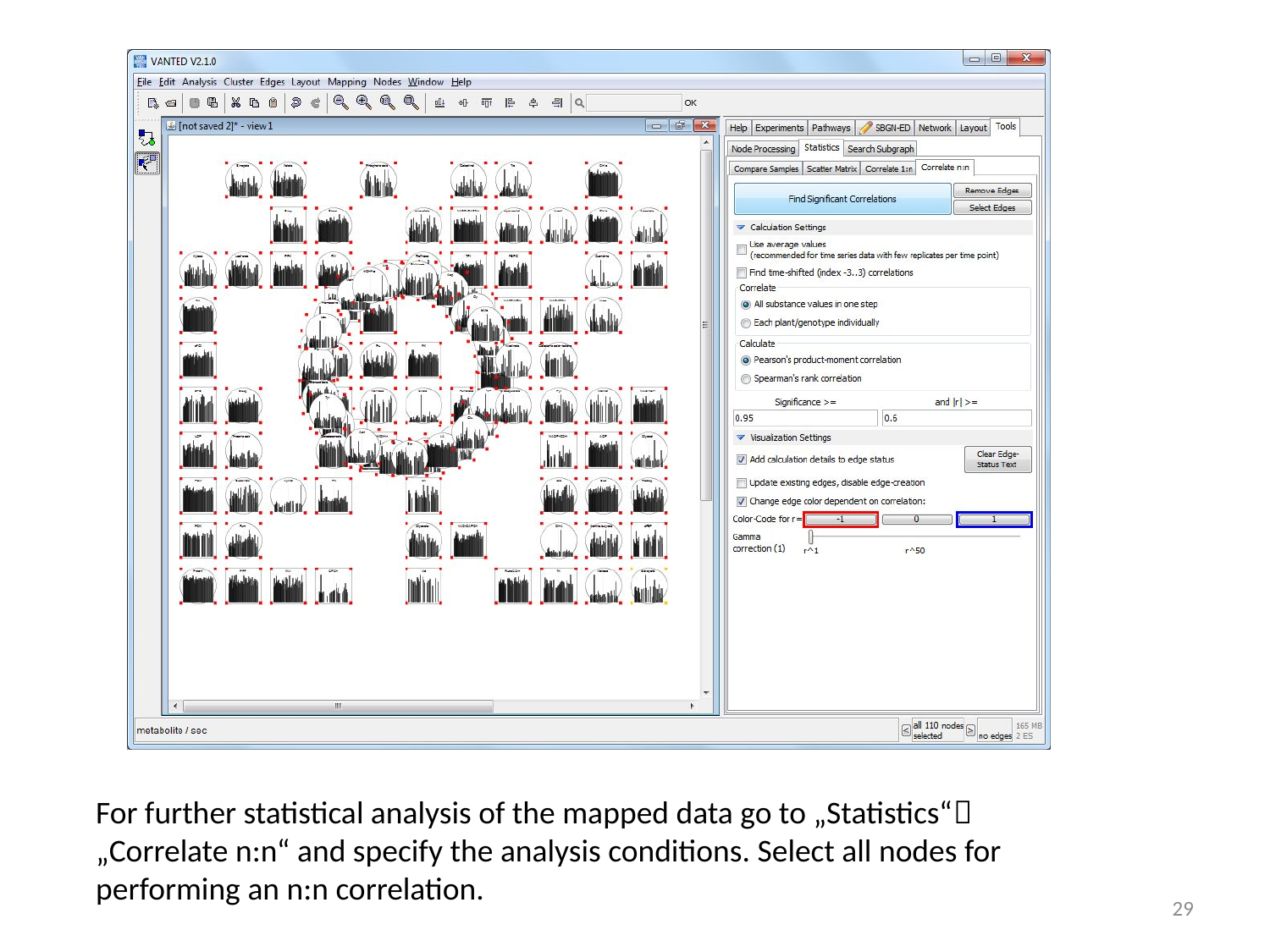

For further statistical analysis of the mapped data go to „Statistics“ „Correlate n:n“ and specify the analysis conditions. Select all nodes for performing an n:n correlation.
29

## Slide 30
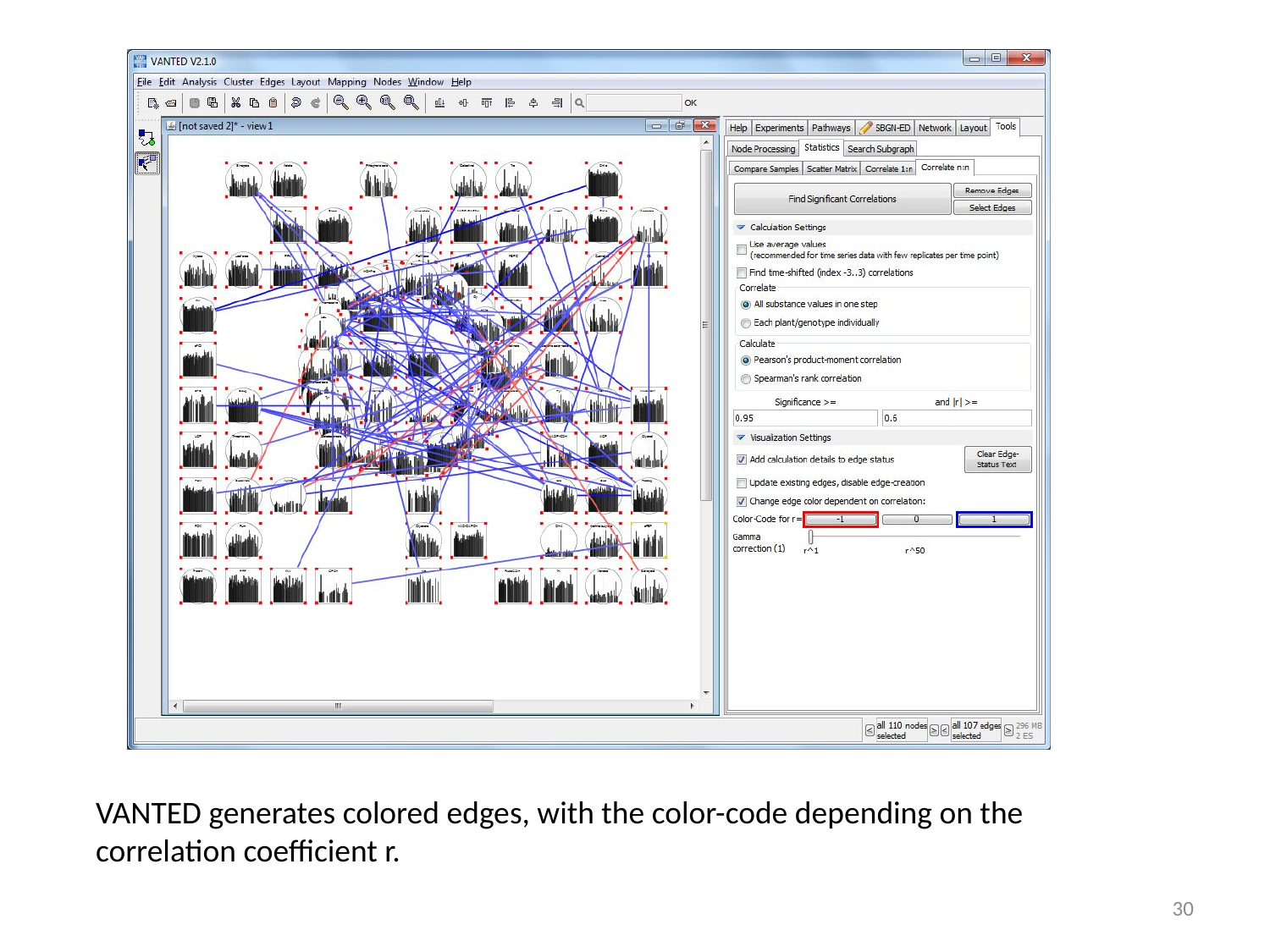

VANTED generates colored edges, with the color-code depending on the correlation coefficient r.
30

## Slide 31
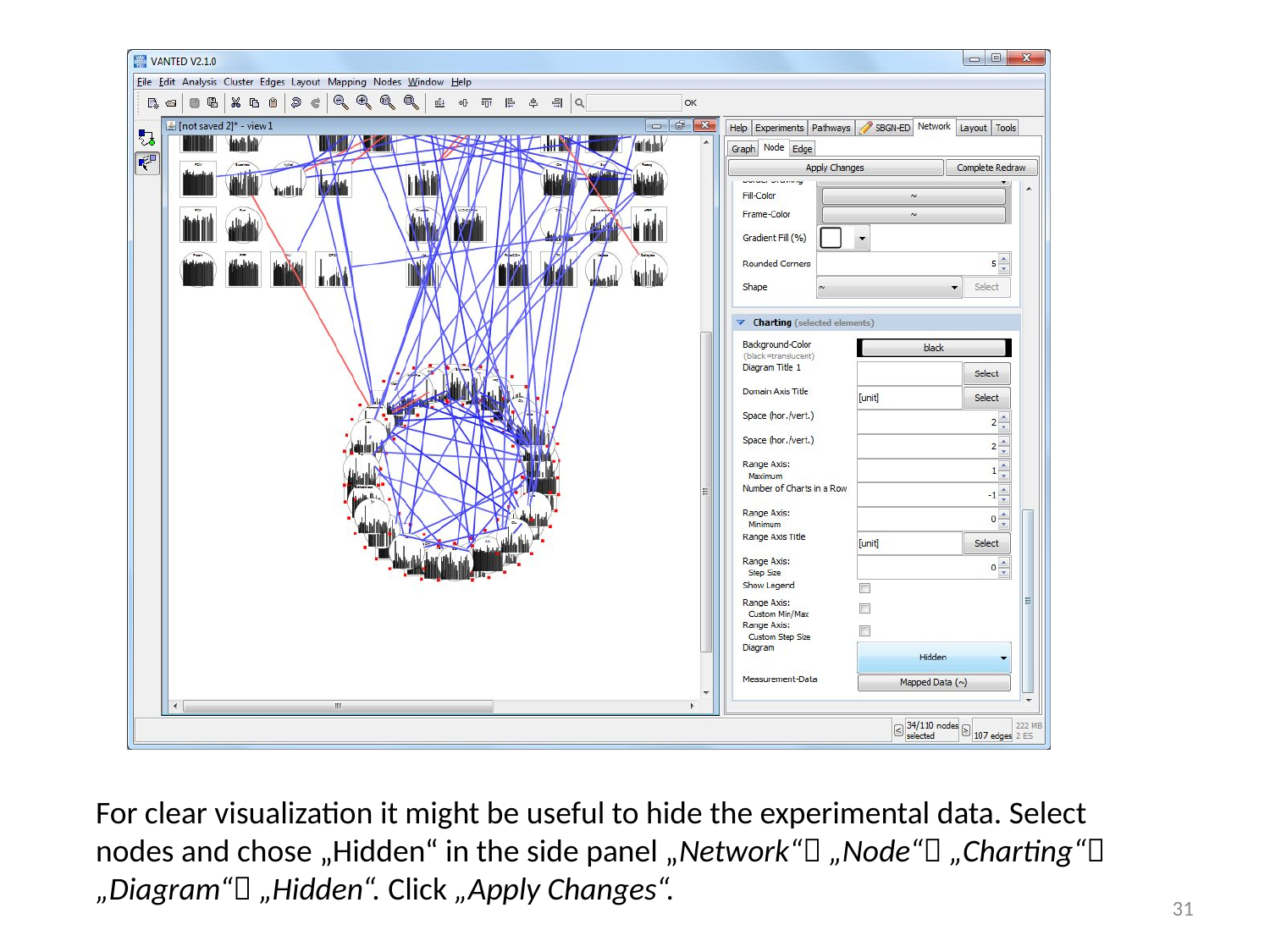

For clear visualization it might be useful to hide the experimental data. Select nodes and chose „Hidden“ in the side panel „Network“ „Node“ „Charting“ „Diagram“ „Hidden“. Click „Apply Changes“.
31

## Slide 32
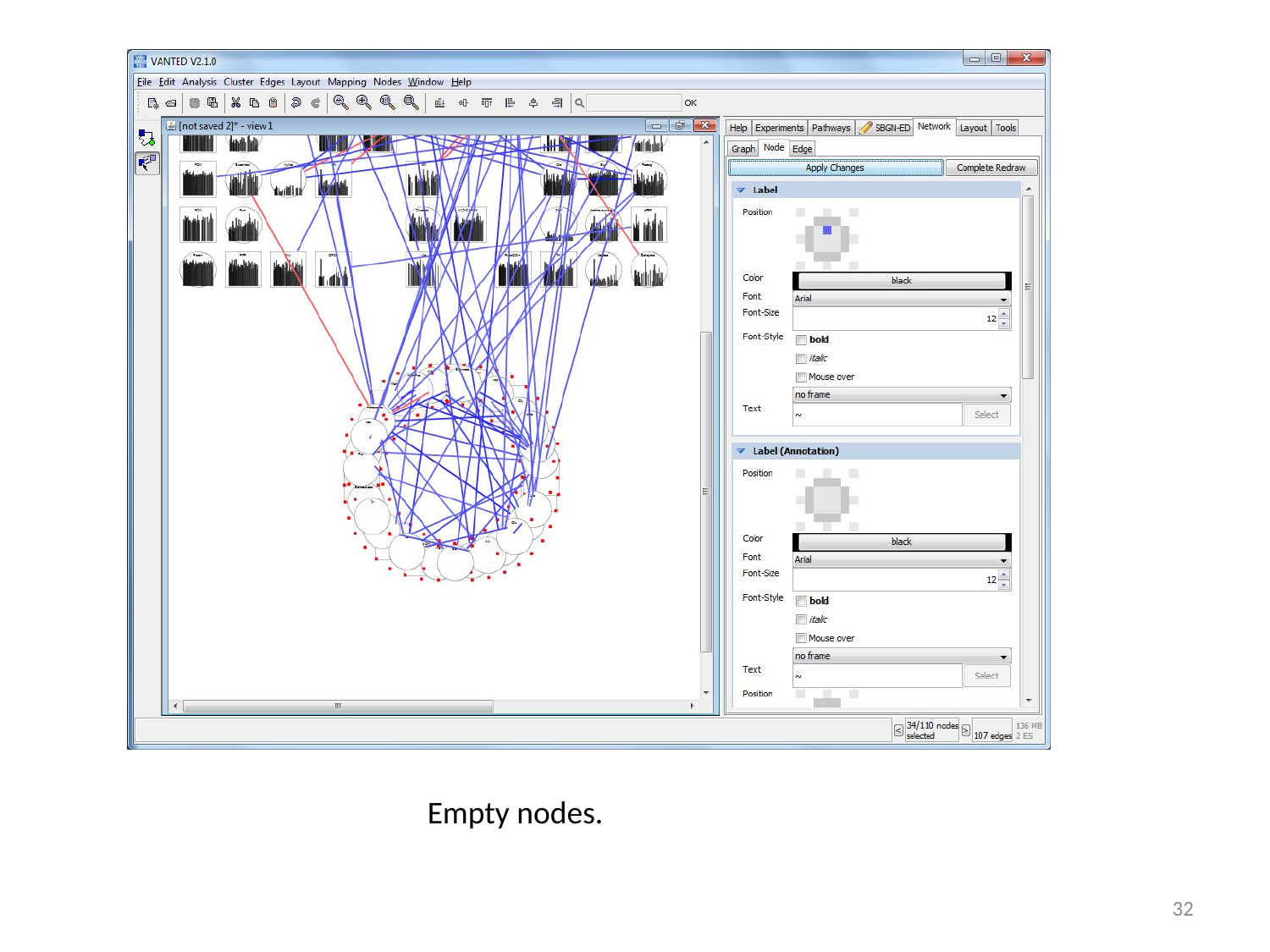

Empty nodes.
32

## Slide 33
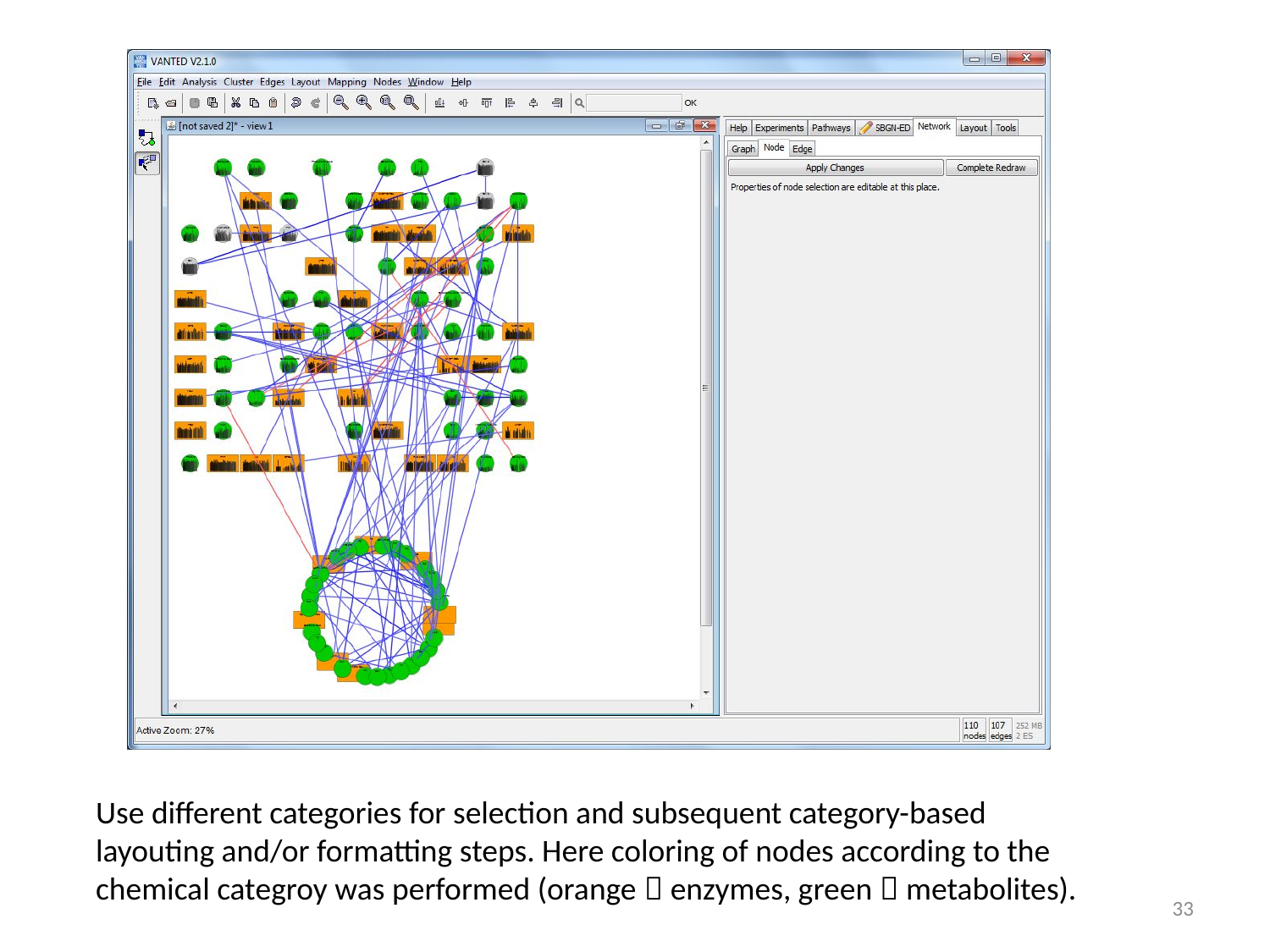

Use different categories for selection and subsequent category-based layouting and/or formatting steps. Here coloring of nodes according to the chemical categroy was performed (orange  enzymes, green  metabolites).
33

## Slide 34
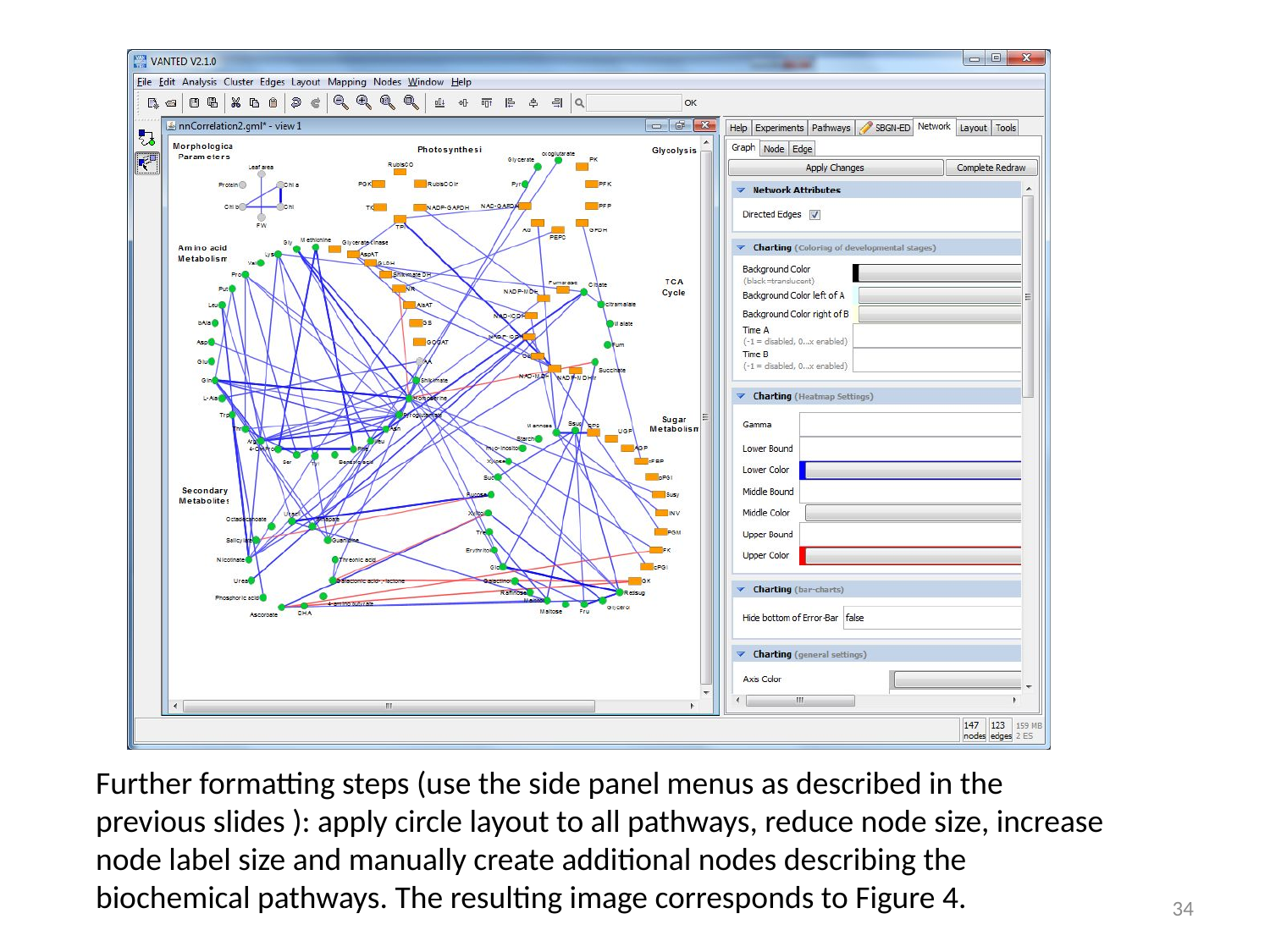

Further formatting steps (use the side panel menus as described in the previous slides ): apply circle layout to all pathways, reduce node size, increase node label size and manually create additional nodes describing the biochemical pathways. The resulting image corresponds to Figure 4.
34
